# BMPR2 affects valve development via ECM–receptor interaction in zebrafish

**DOI:** 10.3389/fcell.2026.1652622

**Published:** 2026-03-23

**Authors:** Yan Shi, Yanli Huang, Yu Xia, Yongqing Li, Yuequn Wang, Wuzhou Yuan, Fang Li, Zhigang Jiang, Yu Chen, Ping Zhu, Jian Zhuang, Xiushan Wu, Xiongwei Fan

**Affiliations:** 1 Medical Research Institute, Guangdong Provincial People’s Hospital (Guangdong Academy of Medical Sciences), Southern Medical University, Guangzhou, Guangdong, China; 2 Guangdong Provincial Key Laboratory of South China Structural Heart Disease, Guangdong Cardiovascular Institute, Guangdong Provincial People’s Hospital (Guangdong Academy of Medical Sciences), Southern Medical University, Guangzhou, Guangdong, China; 3 The Laboratory of Heart Development Research, College of Life Science, Hunan Normal University, Changsha, China; 4 Department of Cardiothoracic Surgery, Guangdong Provincial Hospital of Chinese Medicine, The Second Affiliated Hospital of Guangzhou University of Chinese Medicine, Guangzhou, China; 5 Guangdong Provincial Key Laboratory of Pathogenesis, Targeted Prevention and Treatment of Heart Disease, Guangzhou Key Laboratory of Cardiac Pathogenesis and Prevention, Guangzhou, Guangdong, China

**Keywords:** *bmpr2a*, *bmpr2b*, ECM–receptor interaction, mutant zebrafish, valve development

## Abstract

Abnormal cardiac valve development may lead to functional impairment in adulthood. *BMPR2*, a highly conserved receptor of the BMP family, exists in two subtypes (*bmpr2a* and *bmpr2b*) in zebrafish. However, the roles of *bmpr2a* and *bmpr2b* in valve development remain unclear. In this study, we generated three *bmpr2a/b* mutant zebrafish strains, namely, *bmpr2a*- and *bmpr2b*-knockout zebrafish (*bmpr2a* ^*−/−*^ and *bmpr2b* ^*−/−*^, respectively) using CRISPR/Cas9 and *bmpr2a* and *bmpr2b* double-knockout zebrafish (*bmpr2a* ^*−/−*^ *;bmpr2b* ^*−/−*^) according to *bmpr2a* ^*−/−*^ and *bmpr2b* ^*−/−*^ hybridization. Using cardiac function assessment (M-mode), we characterized the cardiac developmental phenotypes of the three zebrafish mutant strains. Transcriptomic profiling (RNA-seq) was combined with whole-mount *in situ* hybridization (WISH) and qRT-PCR to validate gene-expression changes. The results indicated that *bmpr2a* ^*−/−*^, *bmpr2b* ^*−/−*^, and *bmpr2a* ^*−/−*^ *;bmpr2b* ^*−/−*^ mutant zebrafish strains exhibited valve developmental defects at 52 hours post-fertilization (hpf), followed by cardiac contractile dysfunction. RNA-seq revealed upregulation of cardiac markers (*myl9a*, *myl9b*, *tnnc1a*, *cmlc1*, *myl7*, and *nppa*) and valve-related genes (*fn1b*, *has2*, and *nfatc1*), along with the downregulation of *klf2a*, as validated by WISH and qRT-PCR. Pathway analysis identified the ECM-receptor interaction as a key regulatory axis of *bmpr2a/b*-mediated valve development. In this study, we demonstrate that *bmpr2a* and *bmpr2b* cooperatively regulate cardiac contractile function and valve development in zebrafish, providing insights into BMPR2-mediated cardiovascular morphogenesis in humans.

## 1 Introduction

The cardiac valve is a critical structure that ensures unidirectional blood flow and efficient pumping of the heart. Embryonic developmental abnormalities of the cardiac valve often lead to a high incidence of valve disease in adulthood (O'Donnell and Yutzey, 2020). Valve diseases are essentially the “time-delayed effects” of developmental abnormalities. Genetic defects in embryos, signaling imbalances, and hemodynamic abnormalities in adulthood can lead to diseases triggered by mechanical stress, inflammation, or other factors. For example, in a mouse model with *Alk3* deficiency (Lockhart et al., 2014), there was a significant decrease in epicardium-derived cell migration to the mitral valve’s left leaflet, leading to a 16% increase in the volume of the left leaflet during development. This developmental defect results in thickened and elongated valve leaflets and mucoid degeneration during adulthood. Elucidating the intricate relationship between valvular development and disease pathogenesis may enable the development of innovative therapeutic strategies, including early preventive interventions, gene therapy, and mechanical regulation.

Cardiac valve development initiates with the endothelial-to-mesenchymal transition (EndMT) of the endocardial cushions during the embryonic stage, a process regulated by a complex signaling network (Kovacic et al., 2012). EndMT activation is typically triggered by external signals, particularly those mediated by the transforming growth factor-β (TGF-β) superfamily pathway (Bischoff, 2019; Tang et al., 2022). The TGF-β superfamily constitutes a complex pathway comprising more than 30 ligands and receptor molecules, including bone morphogenetic proteins (BMPs). Among BMPs, BMP2 and BMP4 serve as the primary ligands during valve development, binding to the type I receptor Alk3 (BMPR1A) and type II receptor (bone morphogenetic protein receptor type 2; BMPR2) to form a tetrameric complex at the cell membrane. Upon ligand–receptor binding, SMAD1/5/8 undergoes phosphorylation, forming a complex with SMAD4 that translocates into the nucleus to regulate target gene expression. This cascade induces transcription factors such as *Twist1*, *Msx1/2*, and *Snail*, thereby promoting EndMT progression. Notably, myocardial-derived *Bmp2* and endocardial endothelial-derived BMP type 1A receptor (*Bmpr1a*) are indispensable for generating endocardial cushion cells expressing mesenchymal markers, *Twist1*, *Msx1/2*, and *Snail* (O'Donnell and Yutzey, 2020). During atrioventricular (AV) cushion development, myocardium-secreted *Bmp2* promotes *Has2* expression to synthesize cardiac jelly, which is a critical substrate for EndMT initiation (Camenisch et al., 2000). *Bmp4* similarly regulates EndMT in both the AV and outflow tract (OFT) cushions during heart development. Heterozygous *Bmp4* knockout mice have reduced AV cushion size, whereas cardiomyocyte-specific *Bmp4* deletion impairs semilunar endocardial cushion expansion because of insufficient cell numbers (Jiao et al., 2003; McCulley et al., 2008).

BMPR2, a type II receptor for BMP ligands, harbors functionally deficient mutations in clinical samples of pulmonary arterial hypertension (PAH). Genetic analysis revealed the presence of *BMPR2* variants in 10%–40% of sporadic cases and 58%–74% of familial cases (Pfarr et al., 2011). Mouse models have recapitulated the pathology of PAH. For example, pulmonary endothelial-specific *BMPR2* deletion induces characteristic PAH features (Hong et al., 2008), and dominant-negative *BMPR2* expression in pulmonary smooth muscle cells elicits similar phenotypes (West et al., 2004; West et al., 2008). Notably, *Bmpr2* ^*+/−*^ mice (not expressing mutant protein) developed severe hypoxia-induced pulmonary hypertension (PH) compared to *Bmpr2* ^*ΔEx2/+*^ mice (expressing mutant protein with impaired T495 phosphorylation of eNOS) (Frump et al., 2016). While *BMPR2* mutations are well-characterized in PAH, their impact on valve developmental defects remains underexplored. Studies on *Bmpr2* knockout mice have shown that homozygous mutants exhibit perinatal lethality, whereas the heterozygote mutants exhibit AV cushion abnormalities, which lead to atrial septal defects, membranous ventricular septal defects, thickened valve leaflets, and aortic malpositioning—all without myocardial developmental defects (Beppu et al., 2009). However, the molecular mechanisms linking *BMPR2* to valve development are unclear.

Zebrafish are a powerful model for studying congenital heart diseases, including valve disease, because of their transparent embryonic and genetic characteristics (Yang et al., 2024). The zebrafish BMP II receptor family comprises two homologous genes, namely, *bmpr2a* and *bmpr2b*, which encode Bmpr2a and Bmpr2b proteins with 50% and 66% sequence identity to human BMPR2, respectively (Monteiro et al., 2008). Both proteins contain conserved structural domains, an ActRI/ActRII ligand-binding domain, a single transmembrane domain, a kinase domain for type I receptor phosphorylation, and a ∼500-amino acid carboxy-terminal tail (Monteiro et al., 2008). Expression profiling revealed that *bmpr2a* and *bmpr2b* are ubiquitously expressed from the 1-cell stage to the 12-somite stage. Thereafter, *bmpr2a* maintains ubiquitous expression, whereas *bmpr2b* becomes enriched in the anterior–posterior axial regions. By the 23-somite stage, both genes are strongly expressed in the anterior head and tail regions, with *bmpr2b* showing robust expression in the proctodeum. Morpholino-based knockdown of *bmpr2a* or *bmpr2b* disrupts left–right asymmetry during cardiac development (Monteiro et al., 2008).

Genetic studies have shown that the loss of *bmpr2a* (*bmpr2a* ^*−/−*^) affects gametogenesis in male zebrafish, resulting in abundant spermatogonia but limited meiosis (Zhang et al., 2020). In contrast, the loss of *bmpr2b* (*bmpr2b* ^*−/−*^) had no effect on male zebrafish, while *bmpr2b* ^*−/−*^ female zebrafish showed severe reproductive defects with much smaller follicles than those of the control ovaries (Zhang et al., 2020). Despite these findings, the molecular mechanisms by which *bmpr2a* and *bmpr2b* regulate cardiac myocyte differentiation, valve development, and cardiac contractile function remain uncharacterized in zebrafish.

In this study, we first utilized CRISPR/Cas9 genome technology to generate *bmpr2a* and *bmpr2b* knockout zebrafish, and crossbreeding female *bmpr2a* ^*−/−*^ with male *bmpr2b* ^*−/−*^ yielded double-heterozygous progeny (*bmpr2a^+/−^;bmpr2b^+/−^*), whose self-crossing showed embryonic lethality in *bmpr2a* ^−/−^;*bmpr2b* ^*−/−*^ double homozygotes. Phenotypic characterization of single- and double-knockout zebrafish strains revealed developmental abnormalities in cardiac contractile function and valve development. Transcriptomic and functional analyses have identified extracellular matrix (ECM)–receptor interaction signaling as a critical pathway through which *bmpr2a* and *bmpr2b* regulate valve development.

## 2 Materials and methods

### 2.1 Zebrafish lines

The AB strain of wild-type zebrafish was purchased from the Institute of Hydrobiology, Chinese Academy of Sciences, and raised in a standardized zebrafish breeding facility (Beijing Aisheng Technology Development Co., Ltd.) at Hunan Normal University. The animal experimental protocol was approved by the Institutional Ethics Committee of the Guangdong Academy of Medical Sciences (KY2024-847-01) and was performed in accordance with the relevant guidelines and regulations.

The CRISPR/Cas9 gene-editing system was used to generate zebrafish *bmpr2a and bmpr2b* knockout. Exons 8 and 9 of the *bmpr2a* and *bmpr2b* genes were selected as potential target sites, and the website http://crispor.tefor.net/crispor.py was utilized to design Guide RNA. The Guide RNA sequence was then linked to the pUC57 sgRNA backbone plasmid through homologous recombination, yielding pUC57-*bmpr2a*-sgRNA1 and pUC57-*bmpr2a*-sgRNA2, along with pUC57-*bmpr2b*-sgRNA1 and pUC57-*bmpr2b*-sgRNA2, respectively. The Guide RNAs are listed in Supplementary Table S1. Guide RNA was amplified *in vitro* and subjected to *in vitro* transcription experiments (Riboprobe® System-T7 Translation Kit (Promega, P1440) to produce sgRNA. The sgRNA (20 ng/μL) was mixed with Cas9 protein (TrueCut Cas9 v2, Thermo Fisher Scientific, A36499, 300 ng/μL) and injected into zebrafish at the one-cell phase. The positively knocked-out zebrafish were screened in the F0 generation and sequenced to verify the knockout. F1 were obtained from F0 zebrafish mated with wild-type (WT) zebrafish, and F1 were partially sequenced to verify the knockout strain. The sequence and genotype primers are listed in Supplementary Table S2. *bmpr2a and bmpr2b* double knockout zebrafish were hybridized using *bmpr2a* and *bmpr2b* knockouts and identified simultaneously using *bmpr2a* and *bmpr2b* primers. In this study, we used four main genotypes, namely, the WT (*bmpr2a* ^*+/+*^ *;bmpr2b* ^*+/+*^), *bmpr2a* homozygotes (*bmpr2a* ^*−/−*^ *;bmpr2b* ^*+/+*^), *bmpr2b* homozygotes (*bmpr2a* ^*+/+*^ *;bmpr2b* ^*−/−*^), and double homozygotes (*bmpr2a* ^*−/−*^ *;bmpr2b* ^*−/−*^), which were obtained from the self-crossing of double heterozygotes (*bmpr2a* ^+/−^;*bmpr2b* ^*+/−*^).

### 2.2 Quantitative real-time polymerase chain reaction

Quantitative real-time polymerase chain reaction (qRT-PCR) was performed as previously described (Shi et al., 2019). For qRT-PCR analysis of whole embryos, genotyping was conducted using tail biopsies from 48 hours post-fertilization (hpf) zebrafish embryos, while the remaining embryonic tissues were immediately stored at −80 °C for subsequent RNA extraction (each group contained six zebrafish embryos). For qRT-PCR analysis of the heart tissues, intact heart tissues (including the outflow tract and inflow tract) were microdissected from 48 hpf zebrafish embryos. The residual tissues were retained for genotyping, and a minimum of 20 heart tissues were collected from each experimental group.

Following genotyping, samples from the same genotype were grouped, and total RNA was extracted using TRIzol (Invitrogen). The cDNA library was then synthesized according to the manufacturer’s instructions (*TransScript*® One-Step gDNA Removal and cDNA Synthesis SuperMix, AT311-03). Finally, qRT-PCR was performed under standard PCR conditions using SYBR Green PCR Master Mix (TaKaRa). All gene expression levels were standardized to *GAPDH* expression and analyzed using the 2^−ΔΔCT^ Livak method. Each genotype was represented by at least three independent biological replicates. All qRT-PCR primers are listed in Supplementary Table S2.

### 2.3 RNA-seq

The samples were prepared according to the qRT-PCR analysis method for whole embryos. After sample preparation, they were transported to Majorbio for total RNA extraction, cDNA library construction, and RNA-seq analysis. All data analyses were performed on the Majorbio Cloud platform (www.Majorbio.com). Differentially expressed genes (DEGs) were identified using screening criteria with a significance threshold of |log_2_FC| >2.0 and *p* < 0.05. The original data were submitted to the NCBI Sequence Read Archive (SRA) database (SRA number: PRJNA1336002).

### 2.4 RNA probe synthesis and whole-embryo *in situ* hybridization

Reverse transcription-PCR was used to amplify the mRNA sequence of the gene for probe preparation, and the reverse primers were added to the T7 promoter sequences. Digoxigenin-labeled antisense RNA probes were synthesized through *in vitro* transcription using the Riboprobe® System-T7 Transcription Kit (P1440, Promega) and ROCHE DIG RNA Labeling Mix (REF 11277073910, Roche), according to the manufacturers’ instructions. The zebrafish embryos were fixed in 4% paraformaldehyde, treated with a gradient methanol series (25%, 50%, 75%, 85%, 95%, and 100%), and stored in 100% methanol.

Whole-embryo *in situ* hybridization (WISH) was performed as previously described (Oxtoby and Jowett, 1993). In brief, stored embryos (30–50 embryos/tube) were rehydrated in a graded methanol/PBST series, digested in 10 mg/mL proteinase K (PBST), fixed again in 4% paraformaldehyde, and pre-hybridized in hybridization buffer. Subsequently, the pre-hybridization buffer was replaced with fresh hybridization buffer containing digoxigenin-labeled RNA probe (300 ng) and incubated overnight at 65 °C. Furthermore, the embryos were washed in different buffers, blocked with 2 mg/mL BSA and 2% sheep serum, and incubated with pre-adsorbed antibody overnight. The embryos were then stained using MABT and AP substrate chromogenic solution until an optimal signal was obtained (approximately 10 min–50 min). The staining reaction was terminated by washing in several changes of PBS and PBST. Finally, embryos were kept at 4 °C and photographed in 6% methylcellulose using a Leica TL 5000 microscope (Leica, Germany). After the embryos were photographed, they were collected for genotyping.

### 2.5 Heart function analysis

Cardiac function analysis in zebrafish embryos was performed as previously described (Fink et al., 2009). In brief, M-mode was conducted using a high-speed EMCCD camera to capture 10-s movies of zebrafish heart activity at 48 hpf under a ×20 microscope objective. The recorded cardiac motion videos were analyzed using custom heart analysis software (SOHA software) to derive the functional parameters: heart rate (HR), heart period (HP), diastolic interval (DI), systolic interval (SI), diastolic diameter (DD), systolic diameter (SD), and fractional shortening (FS). All data were visualized as scatter point histograms. Following imaging, embryos were collected for genotyping.

### 2.6 Phenotypic analyses of zebrafish

Embryos were incubated at 28.5 °C in Petri dishes containing fish water. To prevent the formation of melanin pigments, PTU (Sigma) was added to fish water at a final concentration of 0.003% at the end of gastrulation. Then, the zebrafish embryos at 48, 72, and 96 hpf were immobilized using 6% methylcellulose and positioned with the abdomen facing upward, and the pericardial cavity and cardiac phenotypes were imaged using an Axiocam (Zeiss) for subsequent analysis.

### 2.7 Western blotting

Intact heart tissues (including the outflow tract and inflow tracts) were microdissected from 48 hpf zebrafish, and the residual body tissues were retained for genotyping. Following genotyping, heart tissues from the same genotype were pooled, with a minimum of 30 hearts per experimental group, for subsequent protein extraction. Each genotype was represented by at least three independent biological replicates.

Total protein was extracted by homogenizing pooled heart tissues in 100 μL radioimmunoprecipitation assay (RIPA) buffer (Beyotime). Proteins were then separated by sodium dodecyl sulfate–polyacrylamide gel electrophoresis (SDS-PAGE) using Future PAGE^TM^ 4%–20% 15-well gels (ACE). Separated proteins were transferred to polyvinylidene fluoride (PVDF) membranes (Millipore), which were subsequently blocked with 5% skim milk (CST) in Tris-buffered saline with Tween-20 (TBST) for 2 h at room temperature. After blocking, membranes were incubated overnight at 4 °C with the following primary antibodies: GAPDH (1:50,000, 60004, Proteintech), Vim (1:1,000, T55134, Abmart), Myl7 (1:5,000, GTX128346, GeneTex), Cdh2 (1:1,000, CST, 13116), Cdh1 (1:1,000, 3195, CST), and Fn1 (1:1,000, ab268020, Abcam). The proteins were then incubated with horseradish peroxidase (HRP)-conjugated anti-rabbit immunoglobulin G (IgG) or anti-mouse IgG (1:10,000; Abmart) for 2 h at room temperature. After washing with TBST, protein bands were visualized using an Immobilon@ Western Chemiluminescent HRP Substrate kit (Millipore). The relative signal densities of the protein bands were quantified using ImageJ (1.51 version, NIH) and normalized to GAPDH to account for variations in protein loading.

### 2.8 Immunostaining

Immunostaining was performed as previously described (Yang and Xu, 2012). The tail biopsies from 48 hpf zebrafish embryos were used for genotyping, while the remaining embryonic tissues were fixed in 4% paraformaldehyde (PFA) at 4 °C and embedded in paraffin. Paraffin-embedded tissues were then sectioned into 2 *μ*m–4 *μ*m cross-sections, which were subjected to immunostaining with a primary antibody against myosin light chain 7 (Myl7, 1:200, GTX128346, GeneTex), goat anti-rabbit IgG (H + L) cross-adsorbed secondary antibody (Alexa Fluor™ 594, A-11012, 1:1000, Thermo Fisher Scientific), and 4′,6-diamidino-2-phenylindole (DAPI, 1:5000, 28718–90-3, Proteintech) for nuclear counterstaining.

The heart sarcomere structure was imaged using a Nikon confocal microscope (AX-NIS-Elements). The width and length of the sarcomere bands were measured based on fluorescence images. Three zebrafish embryos with well-preserved and clearly stained heart tissues were selected for analysis for each experimental group. For each heart sample, the width or length of the Z-disc was measured in at least three distinct myofibrils to ensure statistical reliability.

### 2.9 Valve morphology analysis

The Tg (*flia:GFP*) line (Shi et al., 2020) was crossed with *bmpr2a* ^+/−^;*bmpr2b* ^*+/−*^ to generate double heterozygotes, producing *flia:GFP;bmpr2a^+/−^;bmpr2b^+/−^* offspring, which were then subjected to self-crossing. Zebrafish embryos were maintained at 28 °C until 144 hpf, and fluorescent images were captured and analyzed using a Nikon confocal microscope (AX-NIS-Elements). After obtaining the images, embryonic tissues were collected for genotype identification, and the same genomes were grouped. The left and right valves were delineated with yellow and red dotted lines, respectively, and their diameters were measured using Digimizer software. At least six samples were used per group.

### 2.10 Statistical analysis

For comparisons among more than two groups, the Shapiro–Wilk test was utilized to determine the normality of data distribution, and the Levene’s test was implemented to examine the homogeneity of variances. When parametric assumptions were met, one-way analysis of variance (ANOVA) was performed, followed by Tukey’s *post hoc* test for multiple pairwise comparisons. When parametric assumptions were violated, the non-parametric Kruskal–Wallis test was implemented, followed by Dunn’s *post hoc* test combined with the Benjamini–Hochberg correction to account for multiple comparisons biases. All statistical analyses were performed using biological replicates. Data are presented as the mean ± standard deviation (SD) and were visualized using GraphPad Prism software. Statistical significance was defined as follows: ns, *p* > 0.05; *, *p* < 0.05; **, *p* < 0.01; and ***, *p* < 0.001.

## 3 Results

### 3.1 Construction of *bmpr2a* and *bmpr2b* knockout zebrafish strains

**FIGURE 1:**
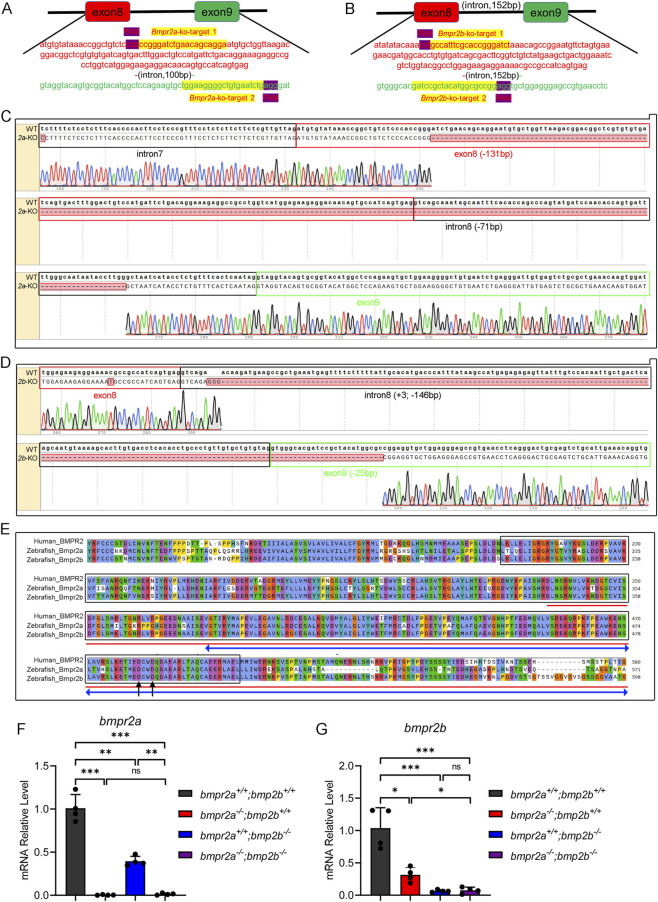
Schematic diagram of *bmpr2a/b* gene knockout in zebrafish. **(A,B)** Schematic diagrams of sgRNA targeting for *bmpr2a* and *bmpr2b* gene knockout, respectively. The red background and font represent exon 8 DNA, the green background and font represent exon 9 DNA, the purple background represents the protospacer adjacent motif (PAM), and the yellow background represents the target sequences. **(C,D)** Alignment between the wild-type and *bmpr2a* and *bmpr2b* knockout genomes using SnapGene software. The red rectangle and font represent exon 8, the black rectangle and font represent intron 8, and the green rectangle and font represent exon 9. **(E)** Protein alignment of human BMPR2 (NP_001195), zebrafish Bmpr2a (NP_001034906), and zebrafish Bmp2b (NP_001034896). The black boxes denote the protein kinase domain; the red lines indicate the deleted sequence of the Bmpr2a protein in the *bmpr2a* knockout sequence; the blue double arrows indicate the deleted sequence of the Bmpr2b protein in the *bmpr2b* knockout mutant; the black arrows (pointing to D482 and D485) indicate two amino acid residues that directly interact with the ligand ACVRL1. **(F,G)** qRT-PCR detected the validation of *bmpr2a* and *bmpr2b* gene knockout efficiency in *bmpr2a* ^*−/−*^, *bmpr2b* ^*−/−*^, and *bmpr2a* ^*−/−*^ *;bmpr2b* ^*−/−*^ mutant zebrafish strains. cDNA was prepared from 48 hpf embryos. The Kruskal–Wallis test was used to compare the statistical significance of differences among the groups. Data are presented as the mean ± SD. *, *p* < 0.05; **, *p* < 0.01; ***, *p* < 0.001; ns, *p* > 0.05.

To characterize the function of BMPR2 in zebrafish, we used CRISPR/Cas9 genome technology to generate *bmpr2a* and *bmpr2b* knockout strains. Bioinformatics analysis using the NCBI and Ensemble databases revealed that both *bmpr2a* (NM_001039817.1, ENSDART00000056764.5) and *bmpr2b* (NM_001039807.1, ENSDART00000125961.3) consist of 13 exons. Guide RNAs were designed to target exons 8 and 9 of *bmpr2a* and *bmpr2b*, respectively, for gene disruption (Figures 1A,B). PCR screening and Sanger sequencing of the F0 generation offspring revealed a 202 bp deletion in *bmpr2a*, including 131 bp within exon 8 and 71 bp in intron 8 (Figure 1C). For *bmpr2b*, mutagenesis resulted in a 171 bp deletion with a 3 bp insertion and a 146 bp deletion in exon 8 and a 25 bp deletion in exon 9 (Figure 1D). Both mutant zebrafish lines exhibited frameshift alterations that were predicted to result in premature translational termination (Figure 1E). Consequently, these mutants lost most of the functional domains of the protein kinase, along with the amino acid residues D482 and D485, which directly interact with ACVRL1, a ligand of the BMP signaling pathway (Iwasa et al., 2023) (Figure 1E).

F1 heterozygotes (*bmpr2a* ^*+/−*^ and *bmpr2b* ^*+/−*^) were generated by crossing F0 founders with wild-type zebrafish, and F2 mutant offspring were obtained by self-crossing of F1 heterozygotes. The results showed that, in both *bmpr2a* and *bmpr2b* mutant zebrafish strains, homozygous offspring from heterozygote self-crosses did not conform to Mendelian inheritance (1:2:1), indicating that the homozygous offspring were partially developmentally lethal (Supplementary Figures S1A, C). The sex ratio of homozygotes of both *bmpr2a* and *bmpr2b* showed severe imbalance; 2 of 57 *bmpr2a* ^*−/−*^ individuals were female in *bmpr2a* ^*−/−*^, and all 71 adult *bmpr2b* ^*−/−*^ individuals were male (Supplementary Figures S1B, D). Zhang et al. (2020) reported that the loss of *bmpr2a* impairs the formation of mature follicles in female zebrafish and spermatogonia meiosis in male zebrafish, whereas *bmpr2b* deficiency disrupts folliculogenesis (resulting in infertility in mutant females) in female zebrafish but does not affect the formation of mature spermatogonia in male zebrafish (Zhang et al., 2020). Notably, their study described gonadal hypertrophy and dysfunction in *bmpr2a/b* mutant zebrafish without significant shifts in the sex ratio. However, we observed marked sex-ratio imbalances in both *bmpr2a* and *bmpr2b* single mutants. This discrepancy may be attributed to differences in the sgRNA-targeting regions. In the study of Zhang et al. (2020), sgRNAs targeting *bmpr2a* and *bmpr2b* were designed for exons 2 and 1, respectively, both of which are located near the ATG start codon. In contrast, in our study, the sgRNAs for *bmpr2a* and *bmpr2b* target exons 8 and 9, respectively, with both regions located far from the ATG start codon. However, the molecular mechanisms underlying these phenotypic differences require further investigation.

Owing to the pronounced sex-ratio imbalance in *bmpr2a* ^*−/−*^ and *bmpr2b* ^*−/−*^, female *bmpr2a* ^*−/−*^ zebrafish were crossed with male *bmpr2b* ^*−/−*^ zebrafish to generate double heterozygotes (*bmpr2a* ^+/−^;*bmpr2b* ^*+/−*^). Self-crossing of these double heterozygotes and genotyping analyses showed that while all other genotypes adhered to Mendelian inheritance, double homozygotes (*bmpr2a* ^−/−^ *;bmpr2b* ^*−/−*^) were absent in adult populations (0.00%, Supplementary Figure S1F). At 48 hpf, however, *bmpr2a* ^−/−^ *;bmpr2b* ^*−/−*^ individuals comprised 7.50% of the offspring, approaching the expected Mendelian ratio of 6.25% (1/16; Supplementary Figure S1E). These results confirmed the developmental lethality of the double mutants, which is consistent with the findings of Zhang et al. (2020). In their study, a significant increase in mortality was observed in *bmpr2a* ^−/−^ *;bmpr2b* ^*−/−*^ mutants at 30 days post-fertilization (dpf).

Subsequent functional analysis utilized offspring from *bmpr2a* ^+/−^;*bmpr2b* ^*+/−*^ self-crossing, focusing on four genotype groups, namely, WT (*bmpr2a* ^*+/+*^ *;bmpr2b* ^*+/+*^), *bmpr2a* homozygotes (*bmpr2a* ^*−/−*^ *;bmpr2b* ^*+/+*^), *bmpr2b* homozygotes (*bmpr2a* ^*+/+*^ *;bmpr2b* ^*−/−*^), and double homozygotes (*bmpr2a* ^*−/−*^ *;bmpr2b* ^*−/−*^), for phenotypic characterization.

qRT-PCR was performed to assess *bmpr2a* and *bmpr2b*. Compared to that of *bmpr2a* ^*+/+*^ *;bmpr2b* ^*+/+*^, *bmpr2a* transcript levels were reduced by ∼95% in both *bmpr2a* ^*−/−*^ *;bmpr2b* ^*+/+*^ and *bmpr2a* ^*−/−*^ *;bmpr2b* ^*−/−*^ (Figure 1F). Notably, *bmpr2a* expression was also reduced by ∼55% in *bmpr2a* ^*+/+*^ *;bmpr2b* ^*−/−*^, indicating cross-regulation between the two paralogs. Conversely, *bmpr2b* expression was reduced by ∼90% in both *bmpr2a* ^*+/+*^ *;bmpr2b* ^*−/−*^ and *bmpr2a* ^*−/−*^ *;bmpr2b* ^*−/−*^ and by ∼65% in *bmpr2a* ^*−/−*^ *;bmpr2b* ^*+/+*^ (Figure 1G). These results confirmed the successful generation of double-knockout zebrafish and revealed the mutual regulatory interactions between *bmpr2a* and *bmpr2b*. This regulatory crosstalk is consistent with prior observations that individual knockdown of *bmpr2a* or *bmpr2b* induces comparable heart laterality defects, whereas simultaneous knockdown does not increase these phenotypes (Monteiro et al., 2008).

Through gene structure analysis and by demonstrating consistency with previous findings, we successfully generated three mutant zebrafish lines, including two single-knockout strains (*bmpr2a* ^*−/−*^ and *bmpr2b* ^*−/−*^) and a *bmpr2a* ^*−/−*^ *;bmpr2b* ^*−/−*^ double-knockout strain. Phenotypic analysis revealed that homozygous mutants for either *bmpr2a* or *bmpr2b* exhibited partial developmental lethality, while all *bmpr2a* ^*−/−*^ *;bmpr2b* ^*−/−*^ double-homozygous embryos failed to survive to adulthood, highlighting the indispensable and synergistic roles of these genes in zebrafish development.

### 3.2 The loss of *bmpr2a* and *bmpr2b* resulted in cardiac contraction at the early embryonic stage of zebrafish

Given that the loss of *bmpr2b* leads to embryonic development lethality in zebrafish, we investigated whether *bmpr2a* and *bmpr2b* affect heart development and cardiac function. Morphological analysis of cardiac development revealed that cardiac looping abnormalities and pericardial edema occurred not only in the double homozygotes *bmpr2a* ^*−/−*^ *;bmpr2b* ^*−/−*^ but also in single homozygous mutants (*bmpr2a* ^*−/−*^ *;bmpr2b* ^*+/+*^ and *bmpr2a* ^*+/+*^ *;bmpr2b* ^*−/−*^) at comparable frequencies (Supplementary Figure S2). The phenotype of heart looping abnormalities is consistent with that of prior studies, in which *bmpr2a/b* knockdown affected the establishment of left–right asymmetry in zebrafish (Monteiro et al., 2008).

**FIGURE 2:**
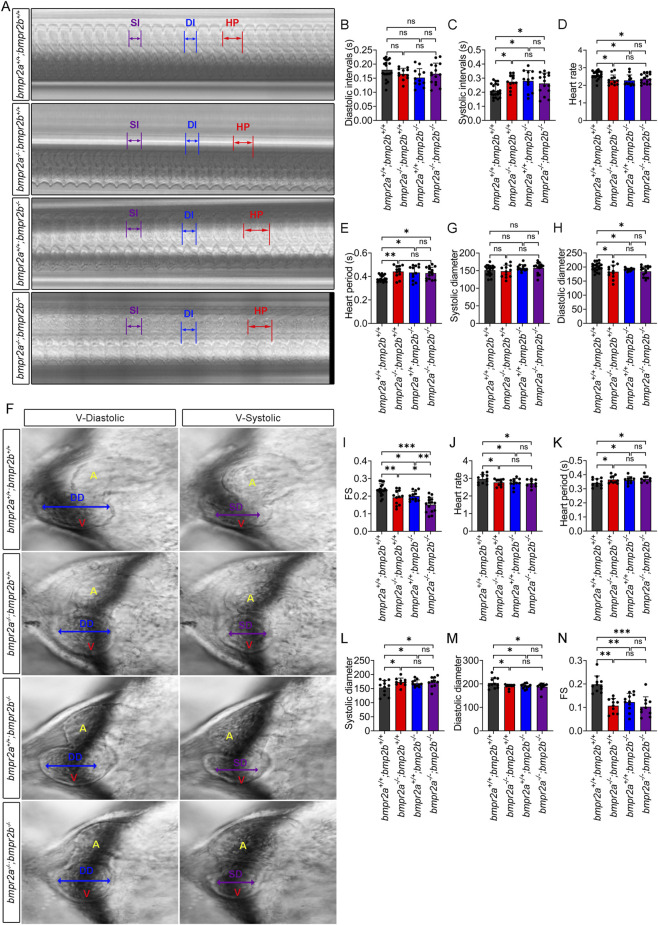
Ventricular morphology and heart parameters analysis via M-mode at 48 and 72 hpf. **(A)** M-modes from movies of embryo hearts at 48 hpf revealed cardiac physiological functions. **(B,C)** Optical recordings of diastolic interval and systolic interval at 48 hpf, respectively. **(D)** Heart rate was measured from the pacemaker activity at 48 hpf. **(E)** Heart period was measured as the interval between the start of one diastole and the beginning of the next at 48 hpf. **(F)** Morphological analysis from the M-mode screenshot displayed the ventricular diastolic and systolic diameter at 48 hpf. **(G)** Statistical results of ventricular systolic diameter at 48 hpf. **(H)** Statistical results of ventricular diastolic diameter. **(I)** Fractional shortening (FS) provides an estimate of the ejection volume at 48 hpf. **(J–N)** Statistical results of the heart rate, heart period, ventricular systolic diameter, ventricular diastolic diameter, and FS at 72 hpf. HP, heart period; SI, systolic interval; DI, diastolic interval; DD, diastolic diameter; SD, systolic diameter; FS, fractional shortening. Data are presented as the mean ± SD (n > 10). One-way ANOVA was used to compare the significance between each group. ns, *p* > 0.05; *, *p* < 0.05; **, *p* < 0.01; ***, *p* < 0.001.

Furthermore, using M-mode (Fink et al., 2009), we analyzed the key parameters of ventricular function at 48 hpf. The results showed that compared to WT controls, *bmpr2a* ^+/+^;*bmpr2b* ^+/+^, *bmpr2a* ^−/−^;*bmpr2b* ^+/+^, *bmpr2a* ^+/+^;*bmpr2b* ^−/−^, and *bmpr2a* ^−/−^;*bmpr2b* ^−/−^ mutant zebrafish exhibited no significant differences in ventricular diastolic interval and systolic diameter (Figures 2A,B,G). However, mutant zebrafish showed prolonged ventricular systolic intervals, altered heart-rate ratios, prolonged cardiac cycles, and reduced ventricular diastolic diameters and fractional shortening (FS) (Figures 2C–F,H,I), indicating impaired contractility and cardiac dysfunction. Collectively, these data indicate that *bmpr2* signaling is critical for cardiac contractile and pacing functions, with loss-of-function mutant zebrafish developing heart failure at 48 hpf. Furthermore, altered heart-rate ratios, prolonged cardiac cycles, increased ventricular systolic diameters, and decreased ventricular diastolic diameters and FS were also observed in mutant zebrafish at 72 hpf (Figures 2J–N). This is consistent with previous research showing that BMP signals, such as BMP2 and BMP4, can regulate the differentiation and formation of sinusoidal node cells, thereby affecting cardiac pacing and the heart rate (Liang et al., 2021; Linscheid et al., 2019; Wang et al., 2023). An irregular heart rate impairs cardiac function through intracellular production of reactive oxygen species (Bergau et al., 2022) and altered expression of sarcomere structure genes and heart failure markers (Lee and Cha, 2021; Sossalla and Vollmann, 2018). Therefore, this may be one of the reasons for diastolic and systolic dysfunction and reduced FS in the three mutant zebrafish strains (*bmpr2a* ^*−/−*^ *;bmpr2b* ^*+/+*^, *bmpr2a* ^*+/+*^ *;bmpr2b* ^*−/−*^, and *bmpr2a* ^*−/−*^ *;bmpr2b* ^*−/−*^).

**FIGURE 3:**
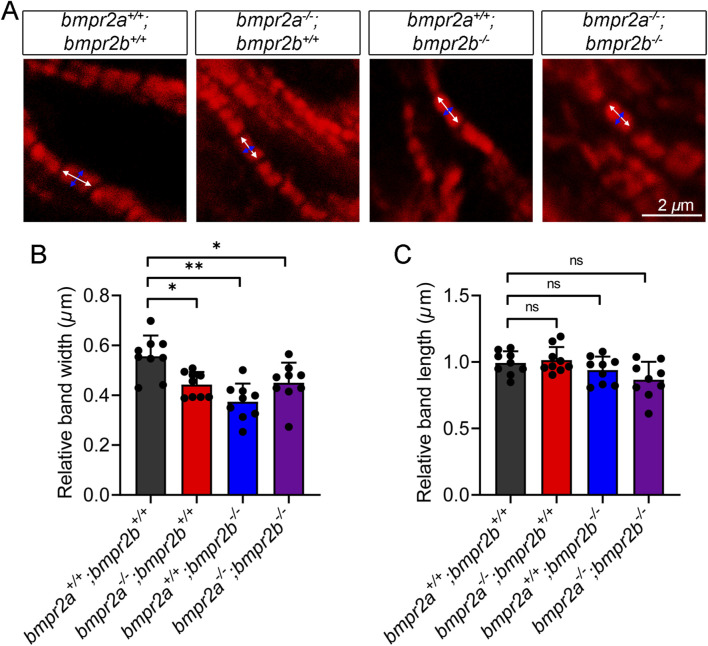
*bmpr2a* ^*−/−*^ *;bmpr2b* ^*−/−*^ affects the myofibril substructures. **(A)** IF-detected subcellular localization of Myl7 exhibited striated bands at 48 hpf. The white bracket lines with double-ended arrows indicate the band length; the blue bracket lines with double-ended arrows indicate the band width. Scale bar, 2 *μ*m. **(B,C)** Measurement of the width and length in A, respectively. Data are presented as the mean ± SD. One-way ANOVA was used to compare the significance between the groups. ns, *p* > 0.05; *, *p* < 0.05; **, *p* < 0.01.

The sarcomere structure is crucial for maintaining the cardiac architecture and enabling myocardial contraction (Zhang et al., 2023). Changes in the sarcomere structure can alter cardiac function (Crocini and Gotthardt, 2021). A previous study demonstrated that the thick filament network of the sarcomere can be visualized via myosin immunostaining (Yang and Xu, 2012). Consistent with this approach, we utilized Myl7 immunostaining to examine the sarcomere structure at 48 hpf in our experimental models. As shown in Figure 3A, sarcomeres in the WT group, *bmpr2a* ^*+/+*^ *;bmpr2b* ^*+/+*^, exhibited an ordered arrangement, and the same structural order was observed in the three mutant groups, namely, *bmpr2a* ^*−/−*^ *;bmpr2b* ^*+/+*^, *bmpr2a* ^*+/+*^ *;bmpr2b* ^*−/−*^, and *bmpr2a* ^*−/−*^ *;bmpr2b* ^*−/−*^ (Figure 3A). Furthermore, comparative analysis revealed that compared with that of the *bmpr2a* ^*+/+*^ *;bmpr2b* ^*+/+*^ group, the width of the *bmpr2a* ^*−/−*^ *;bmp2b* ^*+/+*^, *bmpr2a* ^*+/+*^ *;bmpr2b* ^*−/−*^, and *bmpr2a* ^*−/−*^ *;bmpr2b* ^*−/−*^ embryos was significantly decreased, while the length of the bands was not affected (Figures 3B,C). Although the width of the sarcomere was affected in the three mutant strains, the changes were not sufficient to affect the contraction and relaxation functions of the nervous system; instead, together with the heart ratio, they induced diastolic and systolic dysfunction and reduced FS.

### 3.3 The loss of *bmpr2a* and *bmpr2b* resulted in abnormal valve development in zebrafish embryos

**FIGURE 4:**
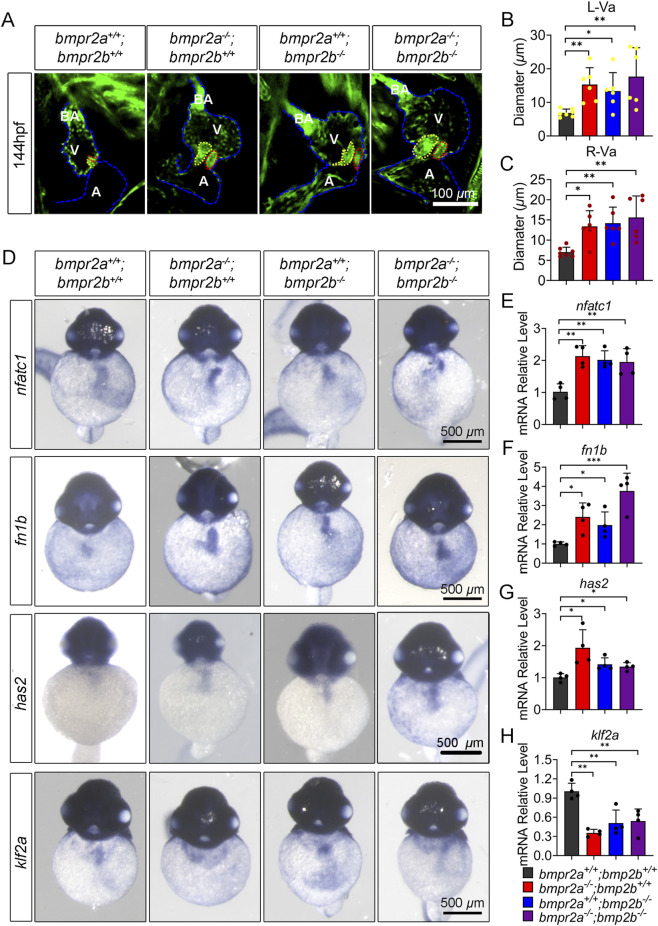
*bmpr2a* ^*−/−*^ *;bmpr2b* ^*−/−*^ affects the valve development. **(A)** Tg (*flia:GFP*) zebrafish line showing the overall structure of the heart at 144 hpf. The red dashed line indicates the heart structure; the blue dashed line indicates the valve structure. A, atrium; V, ventricle; BA, bulbus arteriosus. Scale bar: 100 µm. **(B,C)** Quantification of the left valve and right valve diameter in A. L-Va (left valve) is represented by yellow dotted line in A; R-Va (right valve) is represented by the red dotted line in A. n ≥ 6. **(D)** WISH detected the expression of *nfact1*, *fn1b*, *has2*, and *klf2a* in mutant zebrafish at 52 hpf. **(E–H)** qRT-PCR detected the expression of *nfact1*, *fn1b*, *has2*, and *klf2a* in the mutant zebrafish at 52 hpf. Data are presented as the means ± SD. The Kruskal–Wallis test was used to compare the statistical significance of differences among the groups. ns, *p* > 0.05; *, *p* < 0.05; **, *p* < 0.01.

In zebrafish, the cardiac valve matures into a functional structure at 144 hpf, and the *Tg* (*flia:EGFP*) mutant line, which specifically labels endothelial cells, has been validated as a reliable tool for visualizing the valve architecture (Duchemin et al., 2019). To assess valve structural integrity, we analyzed the *Tg* (*flia:EGFP*) line at 144 hpf. Compared to that in WT (*bmpr2a* ^*+/+*^ *;bmpr2b* ^*+/+*^) zebrafish embryos, the atrioventricular valve of *bmpr2a^−/−^* *;bmpr2b* ^*+/+*^, *bmpr2a* ^*+/+*^ *;bmpr2b* ^*−/−*^, and *bmpr2a* ^*−/−*^ *;bmpr2b* ^*−/−*^ zebrafish embryos exhibited a significantly thickened morphology (Figures 4A–C).

Cardiac valve development in zebrafish is initiated at 52 hpf and is governed by key markers, including *nfatc1*, *fn1b*, *has2*, and *klf2a* (Huang et al., 2018; Steed et al., 2016). To explore the molecular basis of valve developmental defects in *bmpr2*-knockout mutant zebrafish, we performed WISH to assess gene expression at 52 hpf. Compared to that in *bmpr2a* ^*+/+*^ *;bmpr2b* ^*+/+*^ embryos, *nfatc1*, *fn1b*, and *has2* showed significantly upregulated expression in *bmpr2a^−/−^* *;bmpr2b* ^*+/+*^, *bmpr2a* ^*+/+*^ *;bmpr2b* ^*−/−*^, and *bmpr2a* ^*−/−*^ *;bmpr2b* ^*−/−*^ mutant strains (Figure 4D). Conversely, *klf2a* expression was downregulated in all mutant strains (Figure 4D). qRT-PCR validated these findings, demonstrating consistent upregulation of *nfatc1*, *fn1b*, and *has2* and downregulation of *klf2a* in all mutant strain embryos (Figures 4E–H). Collectively, these results indicate that *bmpr2* loss-of-function mutation disrupts the transcriptional program governing valve development in zebrafish.

### 3.4 Transcriptome analysis of differentially expressed genes in *bmpr2a/b* knockout zebrafish

These results indicated that *bmpr2*-knockout mutant zebrafish exhibited abnormal cardiac function and valve development at 48 and 52 hpf, respectively. To uncover the underlying molecular mechanisms, we performed transcriptome sequencing (RNA-seq) on embryos at 48 hpf. DEGs were identified using strict criteria (*p* < 0.05 and FC ≥ 2 or ≤0.5). Compared to the *bmpr2a* ^*+/+*^ *;bmpr2b* ^*+/+*^ group, the *bmpr2a* ^*−/−*^ *;bmpr2b* ^*+/+*^ group showed 753 upregulated and 126 downregulated genes, the *bmpr2a* ^*+/+*^ *;bmpr2b* ^*−/−*^ group had 1,197 upregulated and 234 downregulated genes, and the *bmpr2a* ^*−/−*^ *;bmpr2b* ^*−/−*^ group contained 435 upregulated and 153 downregulated genes (Supplementary Figures S3, 4). In contrast, comparisons between double-knockout strains, *bmpr2a* ^*−/−*^ *;bmpr2b* ^*−/−*^, and single-knockout strains revealed minimal DEGs: *bmpr2a* ^*−/−*^ *;bmpr2b* ^*+/+*^ had nine upregulated and four downregulated genes, whereas *bmpr2a* ^*+/+*^ *;bmpr2b* ^*−/−*^ had one upregulated and 0 downregulated genes (Supplementary Figures S3, 4). Only 21 upregulated and 17 downregulated genes distinguished the two single-knockout lines (Supplementary Figure S3A).

Gene Ontology (GO) enrichment analysis of DEGs showed significant clustering in the cellular component (CC), molecular function (MF), and biological process (BP) categories, including small molecule metabolism, extracellular region, peptidase regulation, and oxidoreductase activity (Supplementary Figures S5A–C). These indicate roles in cellular metabolism, the encoding of secreted proteins or extracellular matrix components, extracellular signal transduction or structural support, and protein degradation. Kyoto Encyclopedia of Genes and Genomes (KEGG) pathway analysis identified enrichment in PPAR signaling, phagosome, vascular smooth muscle contraction, and ECM–receptor interaction (Supplementary Figures S5D–F), linking multiple biological processes, including lipid metabolism, immune response, cardiovascular regulation, cell adhesion/migration, and signal transduction. Surprisingly, in the TGFb signaling pathway, except for the downregulation of *bmp1b* gene expression, other members were upregulated; the target genes of the TGFb signaling pathway, except for *gatad2b*, were also upregulated (Supplementary Figure S6).

**FIGURE 5:**
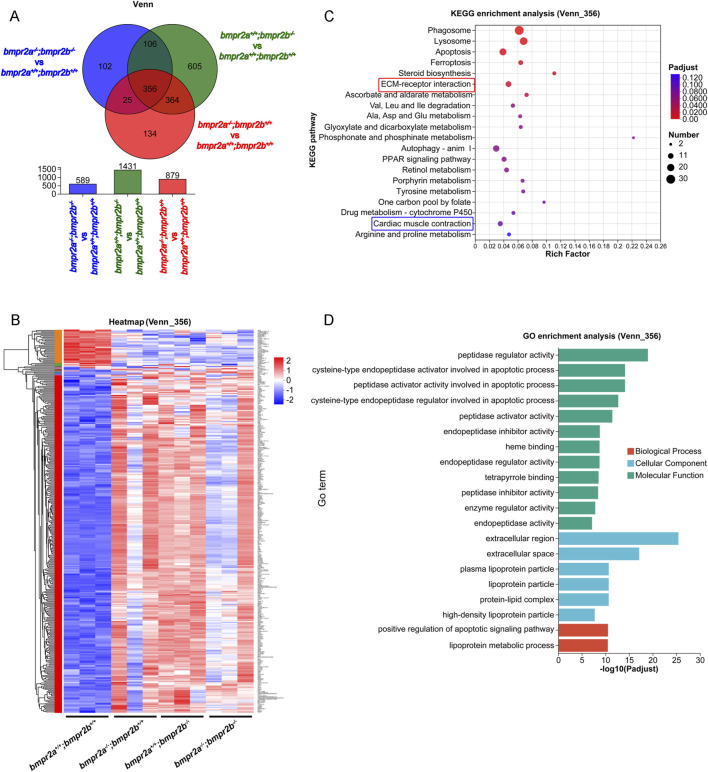
Transcriptomic analysis of differentially expressed genes in *bmpr2a/b* mutant zebrafish. **(A)** Venn analysis of the DEGs of the three groups, namely, *bmpr2a* ^*−/−*^ *;bmpr2b* ^*+/+*^, *bmpr2a* ^*+/+*^ *;bmpr2b* ^*−/−*^ *,* and *bmpr2a* ^*−/−*^ *;bmpr2b* ^*−/−*^. The Venn DEGs were named the Venn_365 subset. **(B)** Heatmap of the Venn_365 subset. **(C,D)** KEGG and GO enrichment analyses of the Venn_365 subset, respectively.

Given the consistent cardiac dysfunction and valve development defects across all three mutant strains, Venn analysis identified 356 commonly dysregulated genes (Figure 5A), which were compiled into the Venn_356 gene subset (Supplementary Table S3).

Heatmap clustering confirmed consistent expression patterns (Figure 5B), with KEGG enrichment in “Cardiac muscle contraction” and “ECM–receptor interaction” (Figure 5C), which are directly relevant to contractile dysfunction and valve development defects, respectively. GO analysis further validated the overrepresentation of the CC, MF, and BP categories (Figure 5D).

**FIGURE 6:**
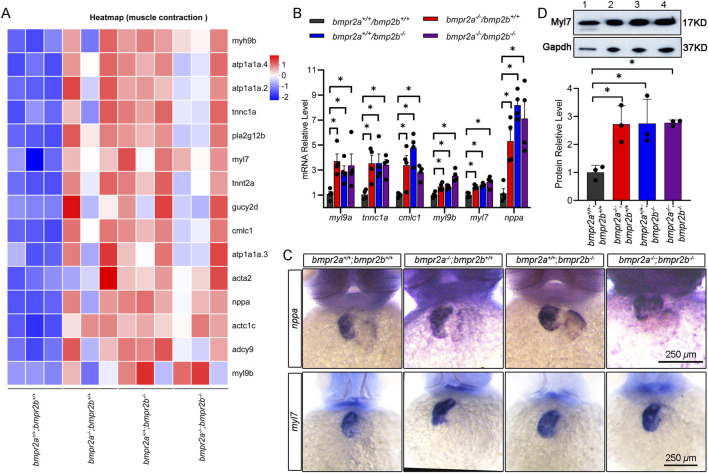
Molecular mechanism verification: *bmpr2a* ^*−/−*^ *;bmpr2b* ^*−/−*^ affects heart contraction at 48 hpf. **(A)** Heatmap of genes that affect cardiac contraction. **(B)** qRT-PCR detected the expression of the cardiac contraction genes. **(C)** WISH verified the expression of the cardiac contraction genes. **(D)** Western blotting detected the expression of the cardiac protein Myl7. Lane 1, wild-type group (*bmpr2a* ^*+/+*^ *;bmpr2b* ^*+/+*^); lane 2, *bmpr2a* ^*−/−*^ *;bmpr2b* ^*+/+*^ group; lane 3, *bmpr2a* ^*+/+*^ *;bmpr2b* ^*−/−*^ group; lane 4, *bmpr2a* ^*−/−*^ *;bmpr2b* ^*−/−*^ group. Data are presented as the mean ± SD. The Kruskal–Wallis test was used to compare the statistical significance of differences among the groups. ns, *p* > 0.05; *, *p* < 0.05.

Functional and molecular analyses converged to show that all three mutant lines (*bmpr2a* ^*−/−*^ *;bmpr2b* ^*+/+*^, *bmpr2a* ^*+/+*^ *;bmpr2b* ^*−/−*^, and *bmpr2a* ^*−/−*^ *;bmpr2b* ^*−/−*^) exhibited heart failure at 48 hpf because of impaired contractility (Figure 2). Transcriptomic analysis revealed consistent upregulation of cardiac contraction genes, validated by qRT-PCR, in both whole embryos and isolated cardiac tissue for cardiac contraction genes *myl9a*, *myl9b*, *tnnc1a*, *cmlc1*, and *myl7* and the heart failure marker *nppa* (Figures 6A,B; Supplementary Figure S7A). WISH for *nppa* and *myl7* showed elevated expression and ventricular dilation trends in the mutant strain compared to those in the WT (Figure 6C). Furthermore, Western blotting analysis using protein extracts from zebrafish cardiac tissue at 48 hpf confirmed that the protein level of *Myl7* was upregulated in the *bmpr2a/b*-knockout zebrafish line (Figure 6D). Collectively, these data demonstrate that the *bmpr2a/b* loss-of-function mutation disrupts cardiac contraction through transcriptional dysregulation of contraction-related genes.

### 3.5 *bmpr2a/b* regulated valve development via the ECM–receptor interaction pathway

**FIGURE 7:**
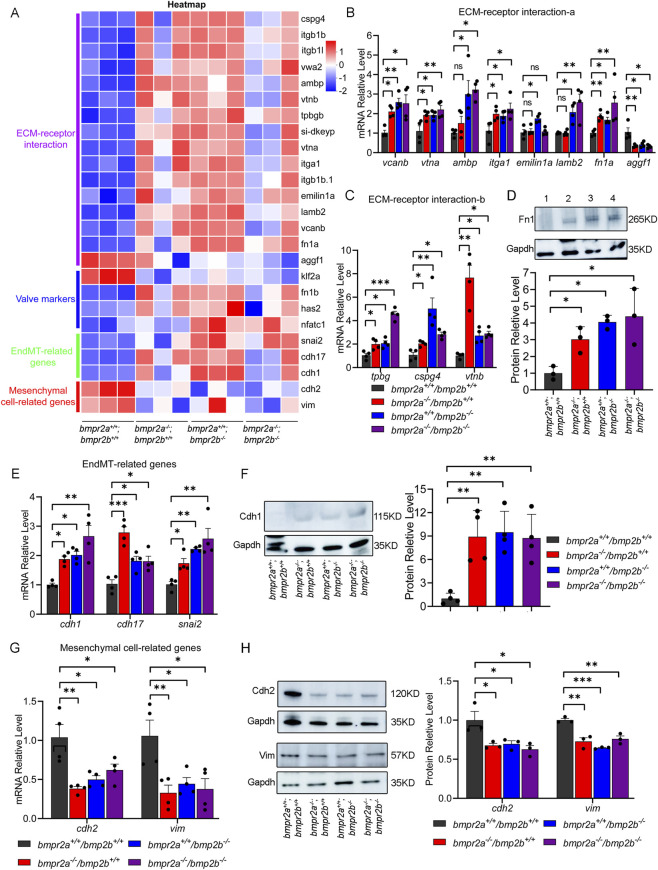
*bmpr2a/b* affects the valve development via the ECM–receptor interaction. **(A)** Heatmap of the ECM–receptor interaction, valve markers, EndMT-related genes, and mesenchymal cell-related genes. **(B,C)** qRT-PCR detected the expression of the ECM–receptor interaction genes. **(D)** Western blotting detected the expression of the ECM–receptor interaction protein Fn1. Lane 1, wild-type group (*bmpr2a* ^*+/+*^ *;bmpr2b* ^*+/+*^); lane 2, *bmpr2a* ^*−/−*^ *;bmpr2b* ^*+/+*^ group; lane 3, *bmpr2a* ^*+/+*^ *;bmpr2b* ^*−/−*^ group; lane 4, *bmpr2a* ^*−/−*^ *;bmpr2b* ^*−/−*^ group. **(E)** qRT-PCR detected the expression of the EndMT-related genes *cdh1*, *cdh17*, and *snail*. **(F)** Western blotting detected the expression of the EndMT-related protein Cdh1. **(G)** qRT-PCR detected the expression of the mesenchymal cell-related genes *cdh2* and *vim*. **(H)** Western blotting detected the expression of the mesenchymal cell-related proteins Cdh2 and Vim. The Kruskal–Wallis test was used to compare the statistical significance of differences among the groups. Data are presented as the mean ± SD. ns, *p* > 0.05; *, *p* < 0.05; **, *p* < 0.01.

The results presented in Figure 4 demonstrate that *bmpr2* insufficiency disrupted valve morphogenesis in zebrafish. This was evidenced by significant alterations in the expression of key valve markers, where *fn1b*, *has2*, and *nfatc1* were upregulated, while *klf2a* was downregulated in all three mutant lines (*bmpr2a* ^*−/−*^ *;bmpr2b^+/+^*, *;bmpr2a^+/+^* *;bmpr2b* ^*−/−*^, and *bmpr2a* ^*−/−*^ *;bmpr2b* ^*−/−*^). These changes in expression were further confirmed by heatmap analysis (Figure 7A).

The ECM is critical for valve remodeling (Kern, 2021), and it is associated with valve disease (Huang et al., 2022). The KEGG enrichment analyses of DEGs between WT and *bmpr2*-knockout zebrafish highlighted significant changes in the ECM–receptor interaction pathway genes across all three mutant zebrafish groups (Figure 5C, red box). Heatmap analysis and qRT-PCR assay conducted on both whole embryos and isolated cardiac tissue consistently demonstrated the upregulation of multiple ECM–receptor interaction-related genes, including *vtnb*, *vtna*, *ambp*, *itga1*, *lamb2*, *fn1a*, *tpbg*, *cspg4*, and *vcanb*, along with the downregulation of *aggf1* (Figures 7A–C, Supplementary Figures S7B, C). Furthermore, Western blotting analysis using protein extracts from zebrafish cardiac tissue at 48 hpf confirmed that the protein level of Fn1 (a key ECM component) was upregulated in the *bmpr2*-knockout zebrafish line (Figure 7D).

Zebrafish valve development initiates at 48 hpf, coinciding with the relative upregulation of ECM (Steed et al., 2016). During ECM remodeling, endocardial cells undergo endothelial-to-mesenchymal transition (EndMT) and subsequent post-EndMT processes, in which mesenchymal cells differentiate into valve interstitial cells, thereby driving valve elongation (Coram et al., 2015). Consistent with these developmental dynamics, our heatmap data showed that *bmpr2a/b*-knockout zebrafish exhibited upregulated EndMT-related genes (*snail*, *cdh1*, and *cdh17*) and downregulated mesenchymal cell-related genes (*cdh2* and *vim*) (Figure 7A). These results demonstrate that the EndMT process is potentially dysregulated during valvular development in *bmpr2a/b*-knockout zebrafish.

**FIGURE 8:**
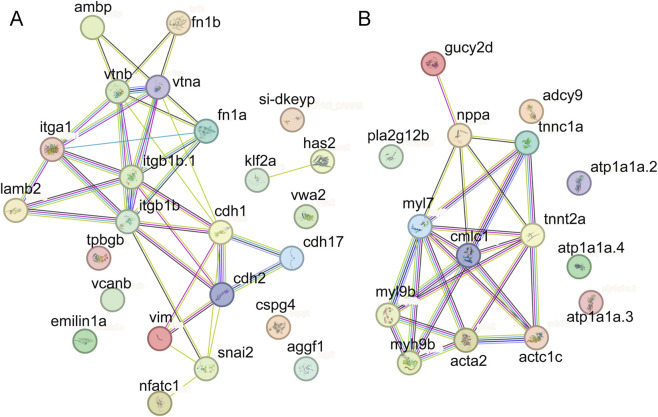
PPI analyses of the regulatory network of valve development genes and cardiac contraction genes. **(A)** PPI analyses of the regulatory network of valve development genes, ECM–receptor interaction genes, EndMT-related genes, and mesenchymal cell-related genes. **(B)** PPI analyses of the regulatory network of cardiac contraction genes. (The PPI website is String: https://cn.string-db.org/cgi/input?sessionId=bVkLDdkSKKje&input_page_active_form=multiple_identifiers).

Although Cdh1 (E-cadherin) is not a canonical EndMT marker, its downregulation is a well-recognized hallmark of cell–cell adhesion loss during both epithelial-to-mesenchymal transition (EMT) and EndMT, reflecting the shift of the cells from a tightly connected epithelial or endothelial phenotype to a highly migratory mesenchymal state (Ma et al., 2020; Singh et al., 2024). Therefore, Cdh1 can be considered an epithelial/endothelial-related gene whose downregulation indicates the progression of EndMT, and we used it to evaluate EndMT dynamics. The above expression pattern was observed in both whole embryos and isolated cardiac tissue (Figures 7E, G, Supplementary Figures S7D, E). Furthermore, Western blotting analysis of protein extracts from zebrafish cardiac tissue at 48 hpf confirmed the transcriptional trends at the protein level; the protein level of the EndMT-related protein Cdh1 was upregulated, whereas the protein levels of Cdh2 and Vim (mesenchymal cell-related genes) were downregulated in the *bmpr2*-knockout zebrafish line (Figures 7F,H). Protein–protein interaction (PPI) network analysis predicted that ECM–receptor genes (*vtnb*, *vtna*, *ambp*, *itga1*, *lamb2*, *fn1a*, *tpbg*, and *cspg4*) act upstream of the EndMT regulators (*snail2*, *cdh17*, and *cdh1*) and mesenchymal-related genes (*cdh2* and *vim*), with the valve marker *nfatc1* positioned downstream of the EndMT effectors (Figure 8A).

**FIGURE 9:**
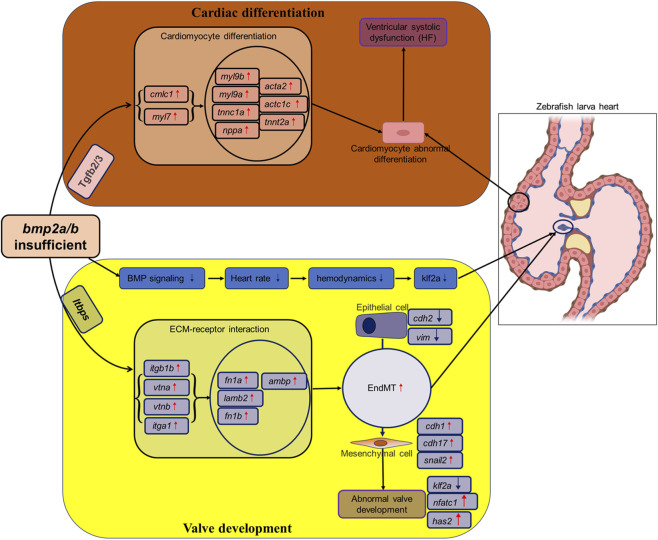
Diagram illustrating the molecular mechanism of *bmpr2a/b* affecting heart looping, cardiac contraction, and valve development; ↓ indicates the genes with downregulated expression in *bmp2a/b-*deficient zebrafish; ↑ indicates the genes with upregulated expression in *bmp2a/b-*deficient zebrafish; the genes enclosed in brackets are the main genes that regulate the expression of the genes in the circles, which were predicted by the PPI regulatory network (Figure 8).

Collectively, these findings revealed that *bmpr2a/b* inactivation upregulated ECM–receptor interaction signaling, which may regulate the EndMT process to facilitate early valve development in zebrafish (Figure 9). However, the specific molecular mechanisms require further experimental verification.

## 4 Discussion

### 4.1 Molecular mechanisms between *bmpr2a/b* and the differentiation of myocardial cells

#### 4.1.1 Sarcomere and heart failure genes regulate cardiomyocyte differentiation

Sarcomere gene dysregulation drives myocardial remodeling, which is a key mechanism implicated in the initiation and progression of heart failure (Marian and Braunwald, 2017). For example, increased myofibrillar density (van Heerebeek et al., 2006) and ACTN2 overexpression (Lan et al., 2025) occur in patients with heart failure and restrictive cardiomyopathy, respectively, while TNNT2 mutations correlate with myocardial remodeling and dilated or hypertrophic cardiomyopathy (DCM/HCM) severity (Ahmad et al., 2008; Li et al., 2021).

For MYL2 (myosin light chain 2), the p.Ile158Thr mutation specifically enhances its expression to induce congenital heart disease (Zhang et al., 2022), and MYL7, which is both a chamber-specific marker and a regulator of heart failure, showed increased phosphorylation following phenylephrine treatment (Grimm et al., 2005). Both Myl7 and the heart failure marker nppa are overexpressed in human cardiac hypertrophy tissues (Newman et al., 2017). In zebrafish embryos, a 7-day exposure to polystyrene nanoplastics (PSNPs) significantly reduced embryo hatching and survival rates, induced cardiac developmental defects, and markedly upregulated *myl7* expression (Liu et al., 2024). During murine cardiac development, *Shh* knockout (*Shh^−/−^*) activates sarcomere genes (including *Myl7*, *cmlc1*, *myl9a*, *myl9b*, *tnnc1a*, and *Acta1*), and the heart failure marker *Nppa* collectively regulates cardiomyocyte differentiation (Rowton et al., 2022).

Consistent with these findings, our three *bmpr2a/b* mutant lines (*bmpr2a* ^*−/−*^ *;bmpr2b* ^*+/+*^, *bmpr2a* ^*+/+*^ *;bmpr2b* ^*−/−*^, and *bmpr2a* ^*−/−*^ *;bmpr2b* ^*−/−*^) exhibited significant upregulation of sarcomere-related genes (*myl7*, *myl9a*, *myl9b*, *tnnc1a*, and *cmlc1*) and the heart failure marker *nppa* (Figure 6). Moreover, the PPI regulatory network identified *myl7* and *cmlc1* as key nodes that may regulate the expression of *myl9a*, *myl9b*, *tnnc1a*, *Acta1*, and *nppa* (Figures 8, 9).

#### 4.1.2 *bmpr2a/b* deficiency induces sarcomere gene activation via *Tgfb2/3*

Both TGF-β and BMP signaling play pivotal roles in myocyte differentiation. BMPR2 mutations impair the differentiation of induced pluripotent stem cells (iPSCs) and intracellular Ca^2+^ transients (Du et al., 2022), whereas BMP signaling, such as BMP10 and BMP2, and BMPR1, promotes cardiomyocyte proliferation during heart development and adult heart tissue regeneration (Sorensen and van Berlo, 2020; Wu et al., 2016). In contrast, TGF-β activation drives actin cytoskeleton reorganization and stress fiber formation (Tzavlaki and Moustakas, 2020); *Tgfb2* is highly expressed in cardiac progenitor cells and cardiomyocytes from 8.5 to 9.5 days in mouse embryos (Dickson et al., 1993), and its deficiency impairs myocardial cell proliferation, leading to ventricular wall thinning (Bhattacharya et al., 2021). *Tgfb3* shows low levels of expression around the outflow tract at 8.5 days in mouse embryos (Dickson et al., 1993), and *Tgfb3* correlates with *NPPA* expression, which is a key factor in dilated cardiomyopathy (DCM) associated with heart failure (Zhu et al., 2022).

Significantly, TGF-β and BMP signaling also exhibit complex synergistic or antagonistic interactions during disease and development. In pulmonary artery smooth muscle cells, BMP inhibition enhances TGF-β signaling and downstream gene expression, whereas *Tgfb1* overexpression suppresses BMP signaling, upregulates *ACTA2*, and promotes osteogenic/adipogenic differentiation of human mesenchymal stem cells (Calvier et al., 2017; Elsafadi et al., 2019). During APAP-induced hepatotoxicity, *BMP7* and *Tgfb1* coordinate tissue repair (Stavropoulos et al., 2022), whereas their expression diverges in human chondrosarcomas (Boeuf et al., 2012).

Our RNA-seq analysis revealed upregulation of *tgfb2* and *tgfb3* in three *bmpr2a/b* mutant lines (compared to *bmpr2a* ^*+/+*^ *;bmpr2b* ^*+/+*^; Supplementary Figures S8A, B), indicating that *bmpr2a/b* deficiency may activate sarcomere genes via TGF-β signaling, especially *tgfb2* and *tgfb3* (Figure 9). However, further functional studies are required to validate this hypothesis.

### 4.2 Molecular mechanisms between *bmpr2a/b* and valve development

#### 4.2.1 *bmpr2a/b* affects valve development via *ltbps*–ECM–receptor interaction-regulated EndMT

ECM–receptor crosstalk is essential for valve development in the heart. Valve development is initiated at 48 hpf in zebrafish, coinciding with the upregulation of ECM-related pathways (Steed et al., 2016). In the ECM microenvironment, two sequential processes, EndMT and subsequent post-EndMT events, are not only critical for driving valve elongation but are also dependent on ECM–receptor crosstalk (Coram et al., 2015). Key ECM components orchestrate valve development by regulating cell adhesion, migration, and tissue remodeling. For instance, *vtna* and *vtnb* (vitronectin isoforms) govern cell adhesion, migration, and tissue remodeling (Leavesley et al., 2013; Peng et al., 2023), whereas *ambp* (alpha-2-macroglobulin precursor) is elevated in calcified aortic valves of patients and high-cholesterol diet-induced *ApoE* ^*−/−*^ mice (Guo et al., 2025). *lamb2* (laminin beta 2) and *fn1* (fibronectin) define the outer ECM signatures of developing tissues (Kremer et al., 2024), with laminin promoting cell proliferation and fibronectin modulating cell death (Chamoux et al., 2002). Notably, aging-induced *lamb2* upregulation in endothelial cells impairs adhesion/migration and enhances EndMT (Wagner et al., 2018), whereas fibronectin (FN) and vitronectin (VTN) collectively regulate endothelial cell dynamics, such as migration and proliferation (Rahman et al., 2005). This evidence further underscores the multifaceted role of ECM components in valve development.

Latent TGF-β binding proteins (LTBPs) act as “escort proteins” that complex with TGF-β and sequester it in the ECM. LTBPs interact with integrin proteins (*itgb1* and *itga1*) to modulate ECM-mediated fibrosis (Hinz, 2015); specifically, Ltbp1 also interacts with fibronectin proteins (*fn1b* and *fn1a*) to immobilize and store TGF-β1 in the ECM, thereby directly regulating ECM stability and elasticity (Klingberg et al., 2018).

In our *bmpr2a/b*-knockout zebrafish models, we observed the upregulation of Ltpbs (*ltbp3* and *ltbp4*, Supplementary Figures S8C, D) and ECM–receptor components (*fn1a*, *lamb2*, *vtna, vtnb*, *itga1*, *itgb1b*, and *ambp*) (Figure 7). This was accompanied by the dysregulation of mesenchymal cell-related genes (*cdh2* and *vim*), EndMT-related genes (*snail2*, *cdh1*, and *cdh17*), and valve markers (*has2*, *klf2a*, and *nfatc1*), indicating abnormal EndMT and valve development (Figures 4, 7).

Furthermore, PPI network analysis indicated that *vtna*, *vtnb*, *itga1*, and *itgb1b* were key regulatory nodes. These nodes potentially govern the expression of downstream targets, including other ECM–receptor components (*fn1b*, *fn1a*, *lamb2*, and *ambp*), mesenchymal cell-related genes (*cdh2* and *vim*), EndMT-related genes (*snail2*, *cdh1*, and *cdh17*), and valve markers (*nfatc1*). Collectively, these data indicate that *bmpr2a/b* regulates ECM–receptor networks via *ltbps*, thereby driving aberrant valve development (Figure 9).

#### 4.2.2 *bmpr2a/b* affects valve development via BMP signaling-mediated modulation of the heart rate

It is worth noting that blood flow serves as a critical regulatory factor in cardiac valve development, promoting valve development by activating the transcription factor *Klf2a* (Fukui et al., 2021). Specifically, during heart valve morphogenesis, the mechanical force generated by blood flow induces the expression of *Klf2a* in endocardial cells. Once activated, *Klf2a* orchestrates downstream signaling cascades to participate in valve remodeling, thereby ensuring proper cardiac valve development. In zebrafish, blood flow-induced *klf2a* expression is significantly upregulated in valve formation regions (such as the atrioventricular canal) (Fukui et al., 2021). Mechanistically, the endocardium senses alterations in blood flow via the mechanosensitive channel *Trpv4*, which, in turn, controls the transcriptional activation of *klf2a* (Gálvez-Santisteban et al., 2019).

Notably, cardiac blood flow dynamics are closely linked to heart rate, which is regulated by the cardiac conduction system. The sinoatrial node (SAN), the primary pacemaker of the conduction system, relies on BMP signaling for its development and function. During the formation of the cardiac conduction system, SAN cells exhibit markedly higher expression levels of BMP signaling components (including the ligands BMP2/BMP4 and the downstream transcription activator SMAD9) than the working cardiomyocytes; these components also co-localize with core SAN marker genes such as *Tbx3*, *Shox2*, and *Hcn4* (Linscheid et al., 2019). Functionally, *Bmp2* acts synergistically with the transcription factors *Shox2* and *Tbx3* to regulate the expression of pacemaker-related genes (e.g., *Hcn4* and *Cacna1d*), which are essential for maintaining the spontaneous electrical activity of SAN cells (Liang et al., 2021). Consistent with this finding, *in vitro* studies have shown that the cardiac mesoderm stage of human induced pluripotent stem cell (hiPSC) differentiation strongly biases cells toward a SAN-specific transcriptional profile, thereby enhancing pacemaker cell specification (Liu et al., 2020). Moreover, overexpression of *Bmp4* alone is sufficient to induce differentiation of hiPSCs into SAN-like pacemaker cells (Wang et al., 2023).

Emerging evidence also indicates crosstalk between BMP signaling and *klf2a* during cardiac development. During zebrafish embryogenesis, *pou5f1*, a transcription factor required for the expression of Klf2/4 family members (*klf2a*, *klf2b*, and *klf17*), cooperates with the BMP signaling pathway to activate and maintain *klf2a* and *klf2b* expression (Kotkamp et al., 2014). Additionally, endocardial cell proliferation is co-regulated by both blood flow and BMP signaling, whereas the hemodynamically sensitive transcription factor *klf2a* contributes to the regulation of endocardial cell morphology (Dietrich et al., 2014).

In this study, all three *bmpr2a/b* mutant genotypes (*bmpr2a* ^*−/−*^ *;bmpr2b* ^*+/+*^, *bmpr2a* ^*+/+*^ *;bmpr2* ^*−/−*^, and *bmpr2a* ^*−/−*^ *;bmpr2b* ^*−/−*^) exhibited reduced heart rate (Figure 2), accompanied by downregulation of *klf2a* expression (Figures 4B,H, 7A). These observations collectively indicate that valve malformations in *bmpr2a/b* mutants may be caused by a secondary decrease in heart rate mediated by a potential BMP signaling–heart rate-hemodynamics–*klf2a* regulatory axis (Figure 9). However, further experimental validation, such as rescue experiments (e.g., restoring heart rate or *klf2a* expression in mutants) or mechanistic studies (e.g., assessing BMP-dependent regulation of pacemaker genes in relation to hemodynamic changes), will be necessary to confirm this hypothesis.

## Data Availability

The data supporting the findings of this study are available in online repositories and supplementary materials. The names of the repository/repositories and accession number(s) can be found below: NCBI Sequence Read Archive (SRA) database, accession number: PRJNA1336002.
