# Supplementary material for: BMPR2 affects valve development via ECM–receptor interaction in zebrafish
Source: Front Cell Dev Biol. 2026 Mar 23;14:1652622. doi: 10.3389/fcell.2026.1652622 (PMC13050836; doi:10.3389/fcell.2026.1652622)
Supplement: Supplementary file 2 [file DataSheet1.docx]

# Supplementary Figure


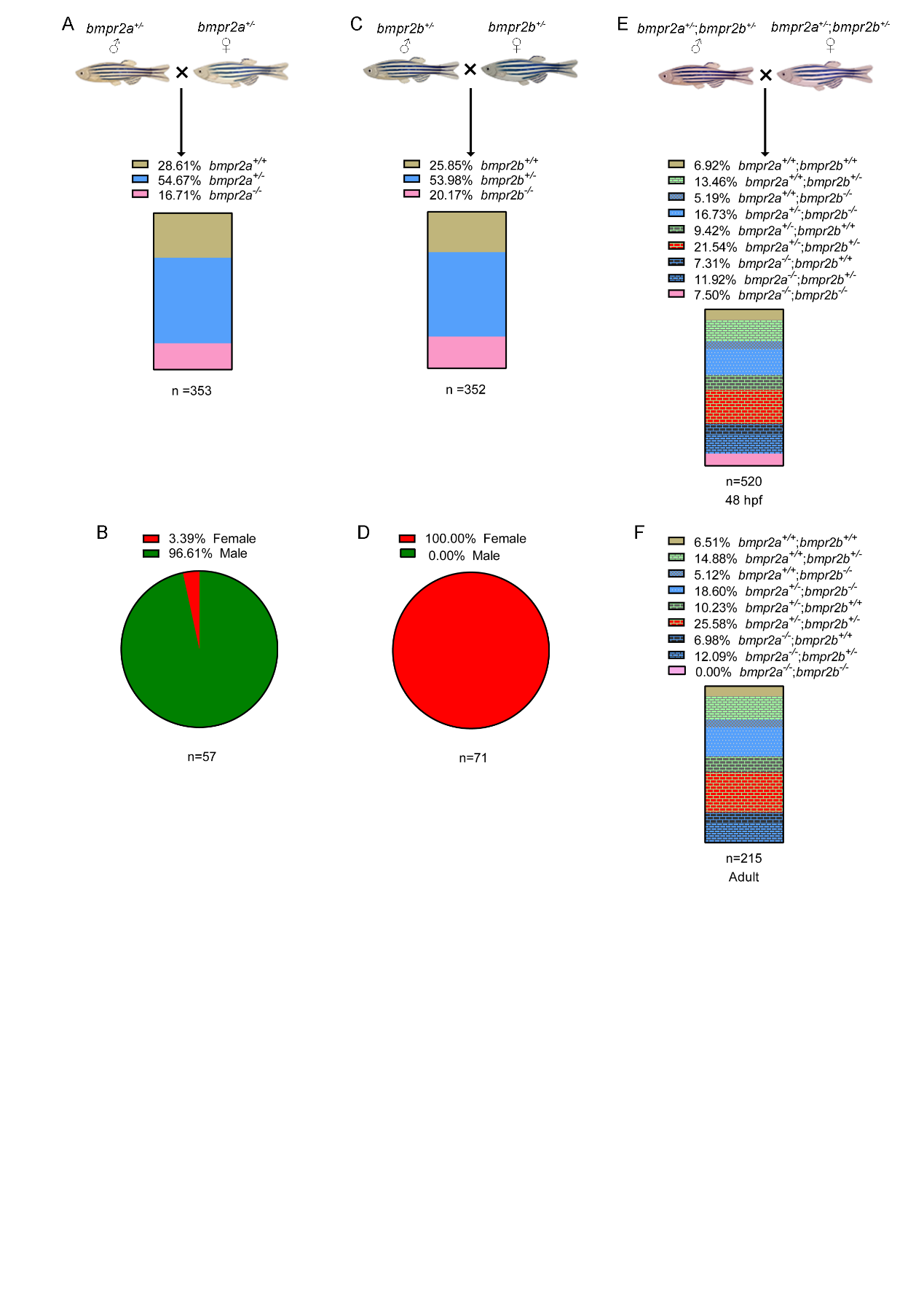


# Figure S1 genotype offspring of bmpr2a/b transgenic zebrafish

**(A)**, the diagram and gene-type ratio at 2-3 month of *bmpr2a^+/-^* offspring. The genotyping (F2) of *bmpr2a^+/-^* (from F1) self-crossing offspring revealed wild-type (*bmpr2a^+/+^*), heterozygous (*bmpr2a^+/-^*) and homozygous (*bmpr2a^-/-^*) genotypes at frequencies of 28.61%, 54.67%, and 16.71%, respectively, which Mendelian inheritance prediction is a 1:2:1 ratio. The reduced homozygote frequency (vs. 25% expected) indicates partial developmental lethality in *bmpr2a^-/-^* zebrafish. **(B)**, the gender ratio of *bmpr2a^+/-^* offspring. Sex ratio analysis of homozygotes showed severe imbalance, only 2 of 57 *bmpr2a^-/-^* individuals were female. **(C)**, the diagram and gene-type ratio at 2-3 month of *bmpr2a^+/-^* offspring. The genotyping (F2) of of *bmpr2b^+/-^* self-crossing offspring heterozygotes revealed wild-type (*bmpr2b^+/+^*), heterozygous (*bmpr2b^+/-^*) and homozygous (*bmpr2b^-/-^*) offspring at frequencies of 25.85%, 53.98%, and 20.17%, respectively, and the homozygous frequency deviated below the Mendelian expectation (25%), indicating partial developmental lethality in *bmpr2b^-/-^* zebrafish. **(D)**, the gender ratio of *bmpr2a^+/-^* offspring. *bmpr2b^-/-^* zebrafish exhibited sex ratio imbalance, and all 71 adult *bmpr2b^-/-^* individuals were male. **(E)**, the diagram and gene-type ratio at 48 hpf of *bmpr2a^+/-^*;*bmpr2b^+/-^*offspring. We crossed female *bmpr2a^-/-^* with male *bmpr2b^-/-^* to generate double heterozygotes (*bmpr2a*^+/-^;*bmpr2b^+/-^*). Self-crossing of these double heterozygous and genotyping analyses showed that all genotypes adhered to Mendelian inheritance at 48hpf. double homozygous (*bmpr2a*^-/-^*;bmpr2b^-/-^*) were absent in adult populations (0.00%, Figure 2F). At 48 hpf, however, *bmpr2a*^-/-^*;bmpr2b^-/-^* individuals comprised 7.50% of the offspring, approaching the expected Mendelian ratio of 6.25% (1/16, Figure 2E). **(F)**, the gene-type ratio at 2-3 month of *bmpr2a^+/-^*;*bmpr2b^+/-^*offspring. Genotyping analyses fromSelf-crossing of these double heterozygous showed that while all other genotypes adhered to Mendelian inheritance, whereas double homozygous (*bmpr2a*^-/-^*;bmpr2b^-/-^*) were absent in adult populations (0.00%), indicated the developmental lethality of double mutants. n, the total number; hpf, hours of post fertilization.


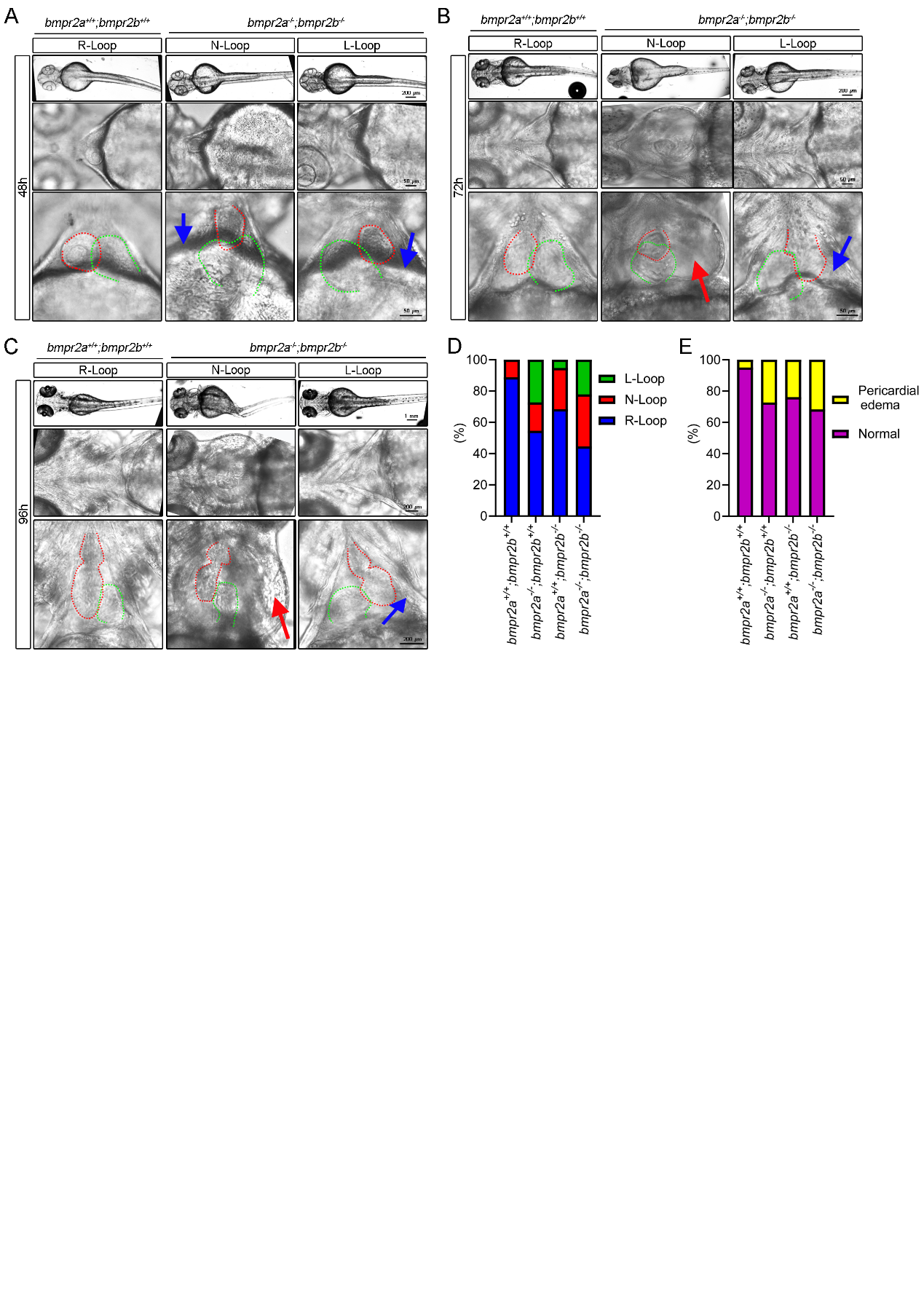


# Figure S2 Heart phenotype analyses in bmpr2a-/-;bmpr2b-/- double-knockout zebrafish

**(A)-(C)**, the heart phenotype of heart looping (normal looping: left looping, L-loop; and abnormal looping, no looping, N-loop, and right looping, R-loop) and edema at 48 hpf, 72 hpf and 96 hpf, respectively. Red and green dot line present the ventricular and atria shape, respectively. The red dotter line indicated the ventricular; the green dotter line indicated the atrium; The blue arrow indicates severe pericardial edema; the red arrow indicates mild pericardial edema. **(D)** and **(E)**, the statistical datu of heart looping and pericardial edema in A-C, respectively.


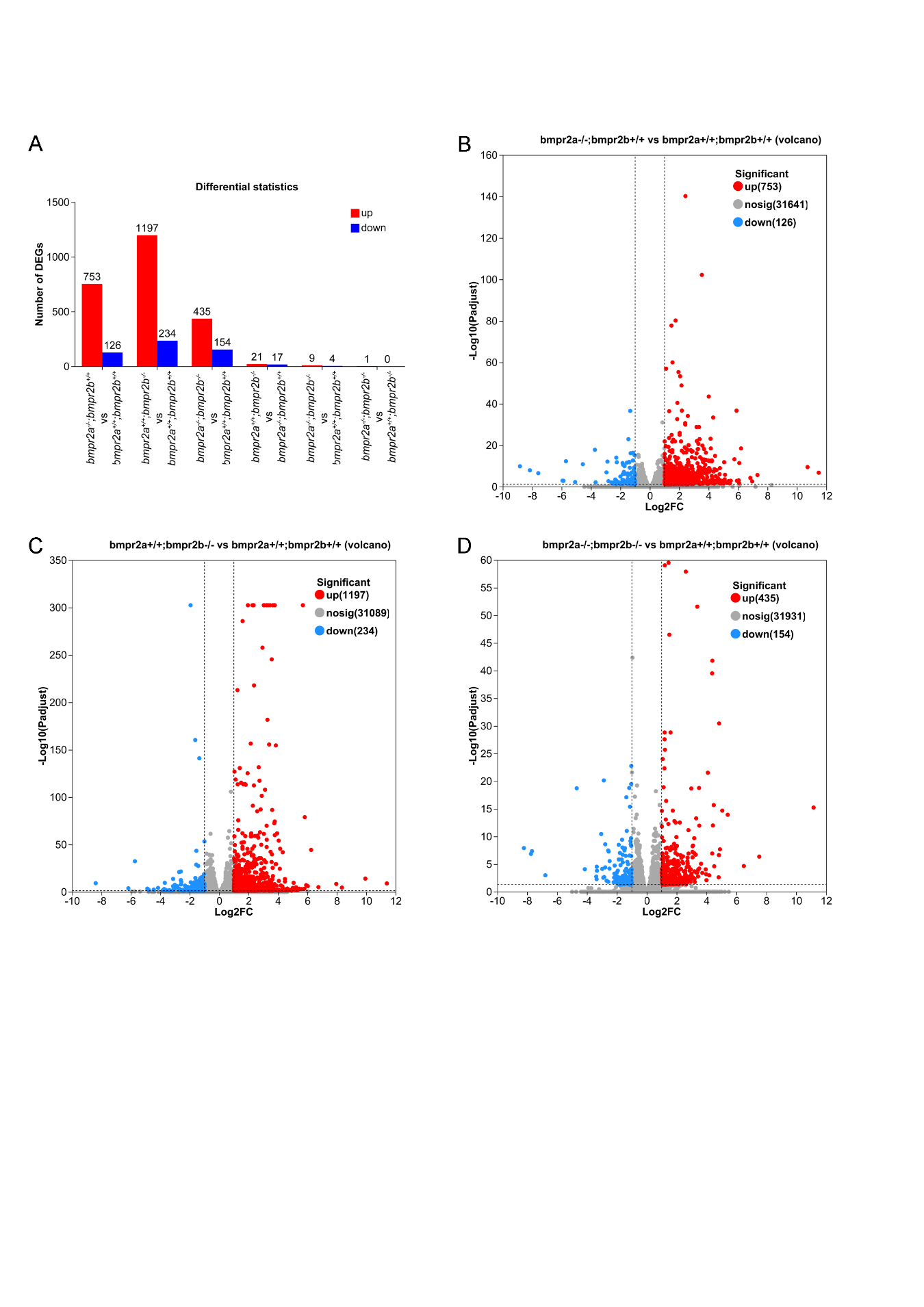


# Figure S3 Transcriptome analysis the differentially expressed genes at bmpr2 transgenic zebrafish at 48hpf

**(A)**, The differential statistics between each two groups.

**(B–C)**, Volcano plot of differentially expressed genes (DEGs). Blue dots represent down regulated genes, red dots represent upregulated genes, and gray dots represent genes with no significant expression differences under different conditions (significance threshold: |log2FC|>2.0 and p<0.05). **(D–F)** Heatmap of DEGs, where rows and columns represent genes and samples, respectively. Red and blue indicate high and low expression levels, respectively. Darker colors denote more pronounced significant differences.


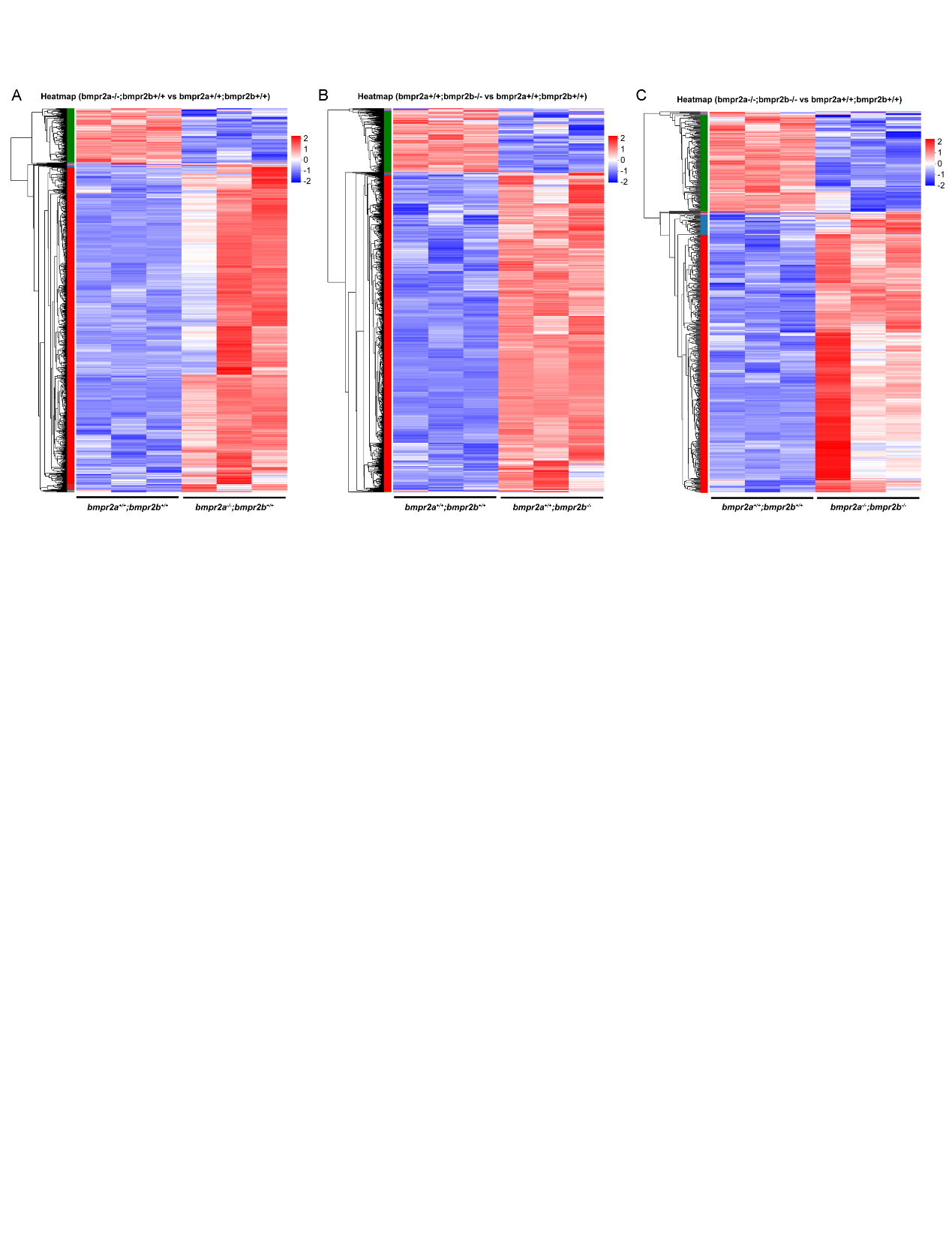


# Figure S4 the heat map of DEGs between wild type group and bmpr2a/b-knockout groups.


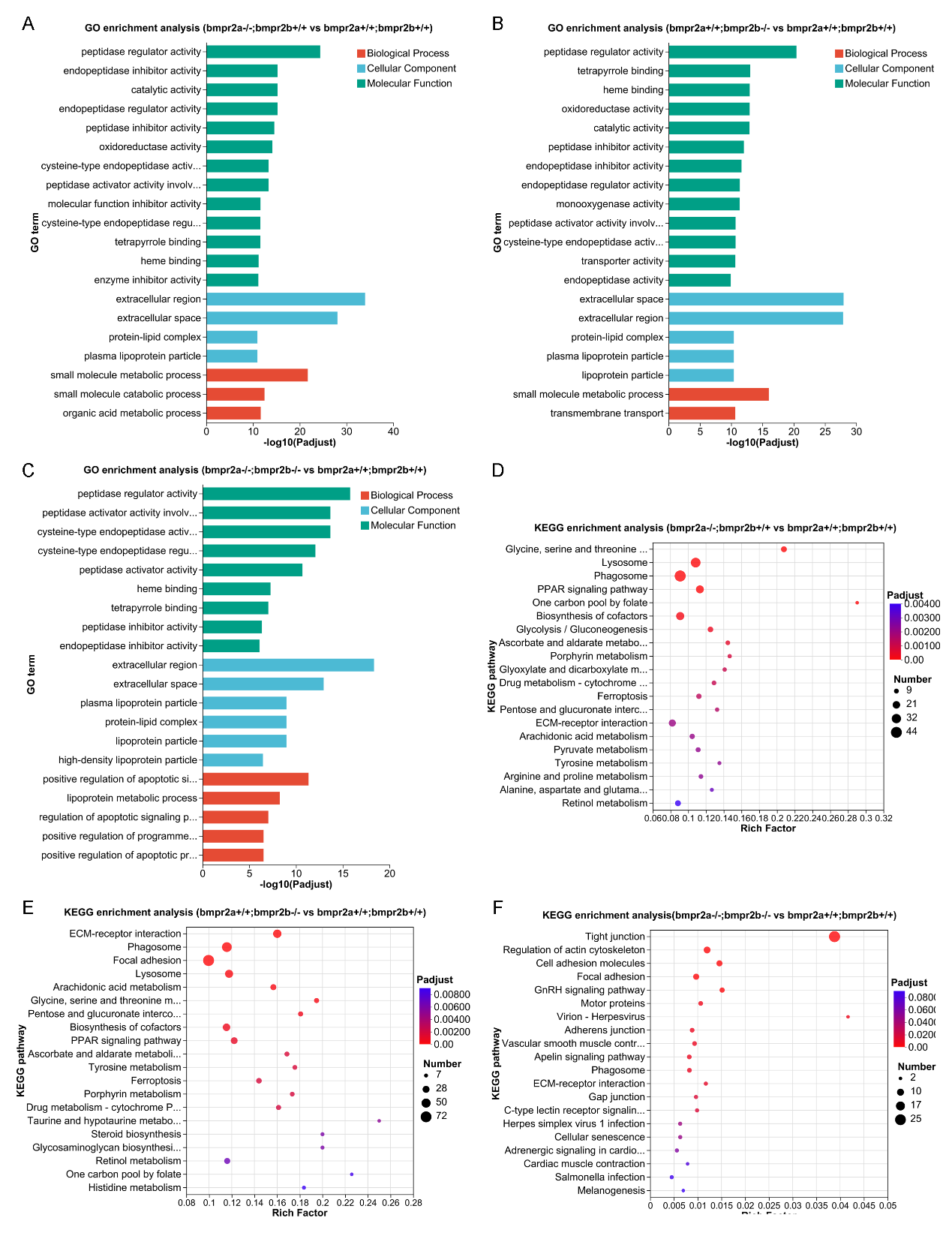


# Figure S5 The Gene Ontology (GO) and Kyoto Encyclopedia of Genes and Genomes (KEGG) pathway enrichment analysis both down-DEGs and up-DEGs.


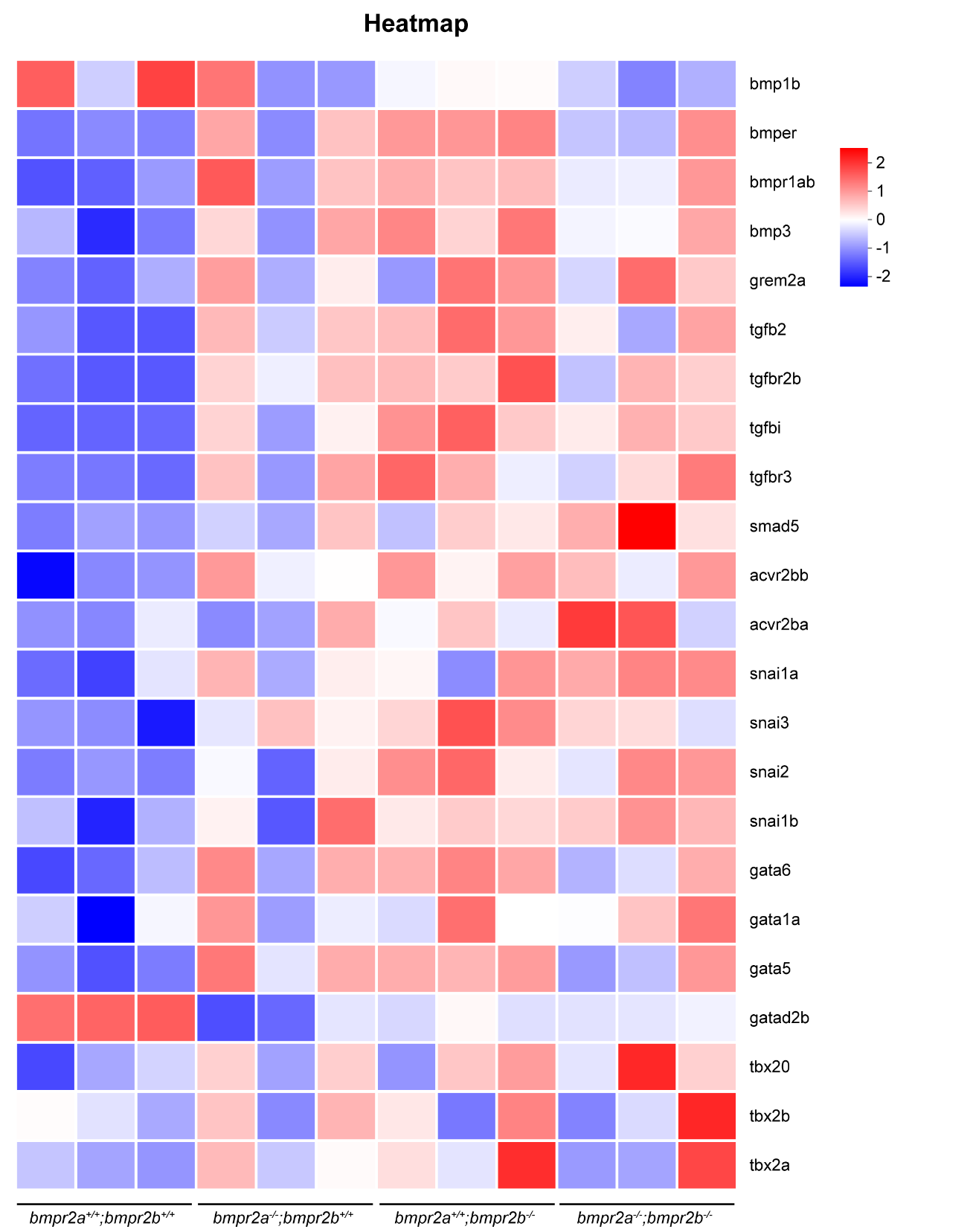


# Figure S6 the heat map of TGFb signaling members and targent genes


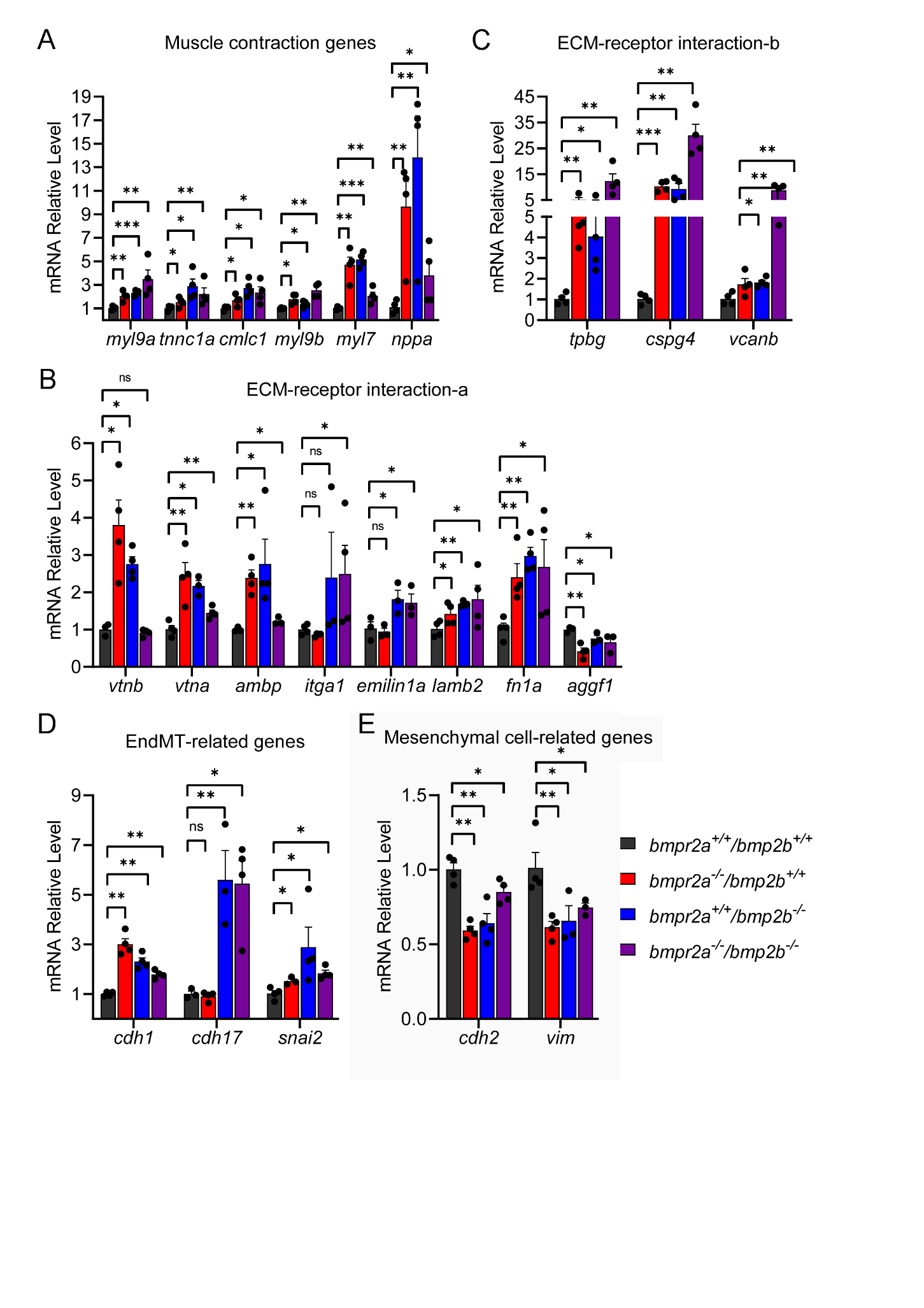


# Figure S7 qRT-PCR detected the expression of different expression genes in the whole embryo at 48 hpf

(**A**), qRT-PCR detected the expression of muscle contraction genes. (**B**) and (**C**), qRT-PCR detected the expression of ECM-receptor interaction genes. (**D**) and (**E**), qRT-PCR detected the expression of EMT markers and mesenchymal cell markers, respectively. Data are presented as means ± SD (n>10). The Kruskal–Wallis test was used to compare the statistical significance of differences among groups. ns, *p*> 0.05; *, *p*<0.05; **, *p*<0.01; *** *p*<0.001.


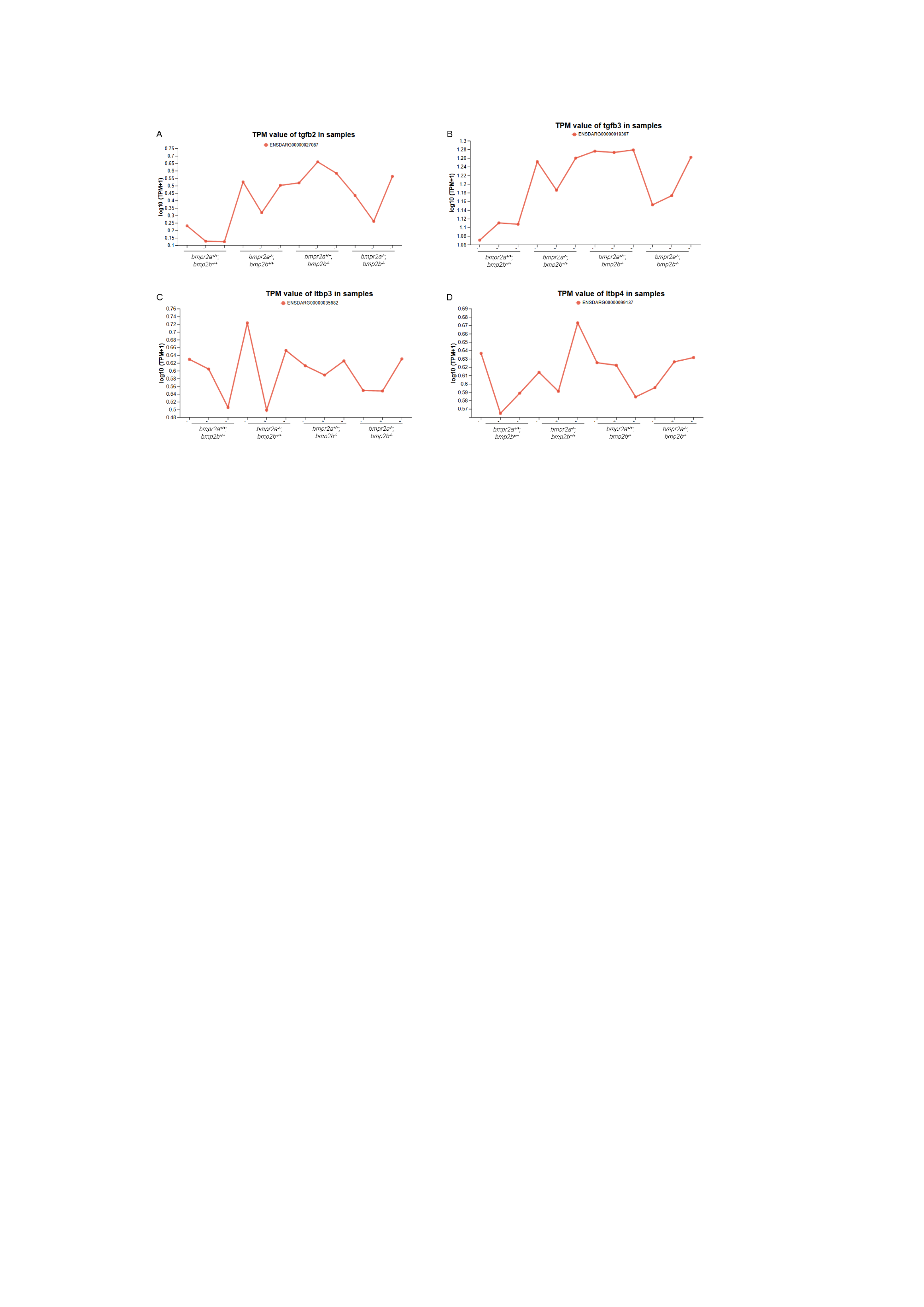


# Figure S8 The TPM of *tgfb2*, *tgfb3*, *ltbp3* and *ltbp4*

TPM, the transcripts (Per Kilobase of exon model) per Million mapped reads/fragments.
